# Abundance and Metabolism Disruptions of Intratumoral Microbiota by Chemical and Physical Actions Unfreeze Tumor Treatment Resistance

**DOI:** 10.1002/advs.202105523

**Published:** 2022-01-17

**Authors:** Fanlei Kong, Chao Fang, Yan Zhang, Lixia Duan, Dou Du, Guang Xu, Xiaolong Li, Hongyan Li, Yifei Yin, Huixiong Xu, Kun Zhang

**Affiliations:** ^1^ Department of Medical Ultrasound and Central Laboratory Ultrasound Research and Education Institute Shanghai Tenth People's Hospital Tongji University School of Medicine No. 301 Yan‐chang‐zhong Road Shanghai 200072 P. R. China; ^2^ Department of Medical Ultrasound Affiliated Hangzhou First People's Hospital Zhejiang University School of Medicine No. 261 Huansha Road Hangzhou 310006 P. R. China; ^3^ Department of Medical Ultrasound and Department of Radiology Guangxi Medical University Cancer Hospital and Guangxi Key Laboratory of Bio‐targeting Theranostics Guangxi Medical University No. 71 Hedi Road Nanning 530021 P. R. China

**Keywords:** flora analysis, inflammation regulation, metabolomics analysis, microbiome metabolism disruption, phototherapy, tumor microenvironment re‐shaping

## Abstract

Intratumoral or intestinal microbiota correlates with tumorigenesis and progression, and microbiota regulation for reinforcing various anti‐tumor approaches is of significant importance, which, however, suffers from no precise regulation method and unclear underlying mechanism. Herein, a microbiome metabolism‐engineered phototherapy strategy is established, wherein Nb_2_C/Au nanocomposite and the corresponding phototherapy are harnessed to realize “chemical” and “physical” bacterial regulations. Flora analysis and mass spectrometry (MS) and metabonomics combined tests demonstrate that the synergistic microbiota regulations can alter the abundance, diversity of intratumoral microbiome, and disrupt metabolic pathways of microbiome and tumor microenvironment, wherein the differential singling pathways and biosynthetic necessities or metabolites that can affect tumor progression are identified. As well, anti‐TNF*α* is introduced to unite with bacterial regulation to synergistically mitigate bacterial‐induced inflammation, which, along with the metabolism disruptions of intratumoral microbiota and tumor microenvironment, unfreezes tumor resistance and harvests significantly‐intensified phototherapy‐based anti‐tumor outcomes against 4T1 and CT26 tumors. The clear underlying principles of microbiome‐regulated tumorigenesis and the established microbiome metabolism regulation method provide distinctive insights into tumor therapy, and can be also extended to other gut microbiome‐associated lesions interference.

## Graphical abstract

A microbiome metabolism‐engineered phototherapy strategy has been established, wherein Nb_2_C/Au nanocomposite and the corresponding phototherapy are harnessed to realize “chemical” and “physical” bacterial regulations via altering the abundance, diversity of intratumoral microbiome, and disrupting inflammation and metabolic pathways of microbiome and tumor microenvironment.

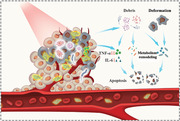

## 1 Introduction

Intestinal microbial diversity and their metabolite diversity determine the pluripotency of gut microbiota.^[^ ^1^ ^]^ An increasing number of records uncovers that microbiome almost pervades across all organs especially in tumors,^[^ ^1^, ^2^ ^]^ and indeed participates in various physiological activities.^[^ ^3^ ^]^ Different medicine communities have witnessed that many diseases, for example, healthy ageing, brain disorders, cardiovascular diseases, etc., are successively found to correlate with gut microbiota and their metabilites.^[^ ^4^ ^]^ In particular, the relevance of symbiotic microbiota and their metabolism to cancer has been comprehensively explored, and massive evidences reveal that intestinal microbiota is closely associated with the origin, progression, and invasiveness of tumors.^[^ ^5^ ^]^ This phenomenon can be attributed to that gut microbiota can bring about tumor metabolism reprogramming through directly secreting microbial metabolites or indirectly altering tumor metabolites,^[^ ^6^ ^]^ because tumor metabolism as well as tumor and microbiota metabolites are demonstrated to correlate with tumorigenesis and directly manipulate tumor progression and evolution.^[^ ^7^ ^]^ More intriguingly, gut microbiota, and their metabolites can remodel immune responses and reshape the susceptibility to various cancer therapies.^[^ ^8^ ^]^ Consequently, they directly interfered with the anti‐tumor outcomes of chemotherapy, radiotherapy, and immunotherapy and thus strengthen the resistances to these therapeutic methods,^[^ ^6^, ^8^, ^9^ ^]^ for example, the intratumoral bacterial can engulf active chemotherapeutic drugs (e.g., gemcitabine) and metabolize them into its inactive form.^[^ ^10^ ^]^ Take all together, gut microbiota modulation using some special method can be regarded as a new pathway to repress tumor.

So far, researches in this field remain at its infancy, for example, what and how molecules or metabolites interfere with tumor progression remain unclear and the underlying metabolism pathway associated with tumor microenvironment is also vague. Moreover, there is no effective and accessible solution of precisely regulating bacteria yet. Nanotechnology has made great progress in tumor diagnosis and treatment due to the high sensitivity, targeting, good compatibility, and high light‐to‐heat conversion efficiency,^[^ ^11^ ^]^ which also furnishes a basis for studying the interaction between bacteria modulation and tumor metabolism.^[^ ^12^ ^]^ More significantly, great advances in nanobiotechnology have driven the progress of many disciplines including postoperative antibiosis and anti‐infection.^[^ ^13^ ^]^

**Scheme 1:**
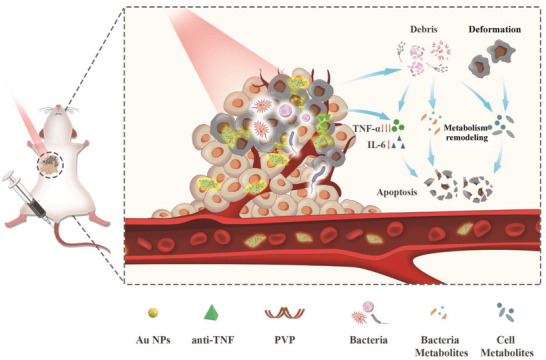
The detailed design principle of antibacterial and anti‐inflammatory actions using such Nb_2_C/Au/anti‐TNF*α*‐PVP nanocomposite to reshape tumor metabolic microenvironment. The “chemical” and "physical synergistic bactericidal effects and phototherapy arising from Nb_2_C/Au could not only kill tumor cells and intratumoral gut microbiome, but they could also modulate the metabolism pathways and alter the abundance and species of metabolites including intratumoral commensal microbiota an tumor cells, wherein the metabolism alteration of intratumoral intestinal microbiota would further contribute to the tailored tumor metabolism microenvironment. As well, the loaded anti‐TNF*α* drug also repressed intratumoral microbiome‐arisen inflammation, which along with aforementioned metabolism modulation of tumor microenvironment, exerted robust anti‐tumor effects against breast tumor.

Enlightened by above experiences, we constructed a specific bacterial‐engineered nanocomposite, based on which a metabolism disruption strategy of intratumoral gut microbiota and tumor microenvironment enabled by phototherapy was developed. This strategy is expected to unfreeze the tumor resistance and repress liver tumor along with direct heat injures and commensal microbiome‐arisen inflammation extinguishment. In this strategy, the specie and abundance of gut microbiota in tumors were altered by this nanocomposite, accompanied with disruptions of tumor metabolism pathway and tumor and microbiota metabolites, during which the inactivation of microbiota‐incurred chronic inflammation was also within easy reach. The outlined mechanism is shown in **Scheme** **1**. The composite is obtained using Nb_2_C nanosheets (NSs) as support to anchor Au nanoparticles (NPs) and accommodate anti‐TNF*α* drug. Nb_2_C NSs as the photothermal agents are designed to produce heat for killing tumor cells and simultaneously decreasing commensal bacterial upon exposure to laser irradiation,^[^ ^14^ ^]^ which are expected to alter tumor and microbiota metabolites for enabling tumor and microbiota metabolism disruptions. As well, the inherent “chemical” anti‐bacterial activity of Au NPs and Nb_2_C NSs are also expected as chemical modulator to reinforce the phototherapy‐incurred metabolism disruption of symbiotic microbiota,^[^ ^15^ ^]^ which will further benefit tumor repression. More significantly, systematic experiments including flora analysis and mass spectrometry (MS) and metabonomics combined analyses demonstrate the feasibility of this microbiota metabolism disruption strategy, wherein the abundance and diversity of microbiota were varied; the differential metabolites and metabolic pathways in both microbiota and tumor differed; and the tumor metabolism microenvironment was re‐shaped. These experiments uncovered the underlying mechanism and answered what and how molecules or metabolites‐composed ecology affect tumor progression.

Besides varying tumor metabolism and metabolites, gut microbiota metabolism disruption is also anticipated to alleviate chronic inflammation,^[^ ^16^ ^]^ which also takes the responsibility for suppressing tumorigenesis and tumor development.^[^ ^17^ ^]^ Inspired by it, anti‐TNF*α* drug that was loaded on Nb_2_C NSs was expected to enable TNF blockading, further inactivate inflammation pathway, decrease inflammatory cytokine and contribute to the chronic inflammation mitigation for attenuating cancer progression (Scheme 1).^[^ ^18^ ^]^ By virtue of the two pathways to mitigate chronic inflammation, such a microbiota and tumor metabolism‐engineered phototherapy is expected to further favor tumor repression.

## 2 Results and Discussion

### 2.1 Gut Microbial‐Engineered Composite (Nb_2_C/Au/Anti‐TNF*α*) Synthesis

**Figure 1:**
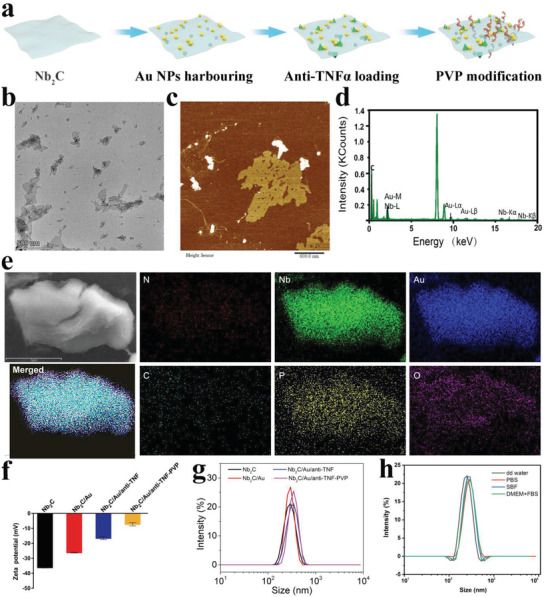
Synthesis and characterizations of Nb_2_C/Au‐anti‐TNF*α*‐PVP and its intermediates. a) Schematic for depicting synthesis procedures of Nb_2_C/Au‐anti‐TNF*α*‐PVP nanocomposite. b,c) Transmission electron microscope (TEM) (b) and atomic force microscopy (AFM) (c) images of 2D Nb_2_C/Au nanosheets; and d) selected area energy disperse spectroscopy (EDS) spectrum of Nb_2_C/Au nanosheets. e) Element mapping images of Nb_2_C/Au/anti‐TNF*α*‐PVP (C, Au, Nb, N, P, and O elements). f,g) Zeta potential and particle size distribution of Nb_2_C/Au‐anti‐TNF*α*‐PVP and its intermediates. Data were expressed as mean ± standard deviation (SD) (*n* = 3). h) Particle size distribution of Nb_2_C/Au‐anti‐TNF*α*‐PVP nanocomposite in different media, for example, double distilled (DD) water, PBS, simulated body fluid (SBF), and Dulbecco's modified eagle medium (DMEM)/fetal calf serum (FBS) mixture.

Intratumoral gut microbial‐engineered composite (i.e., Nb_2_C/Au/anti‐TNF*α*) was obtained after three steps, that is, Nb_2_C nanosheet preparation, Au nanoparticles chelation, and anti‐TNF*α* loading (**Figure** **1**a). The ultrathin Nb_2_C nanosheets with 200 nm lateral size and 1.45 nm thickness are yielded via a classic etching/exfoliation method based on multi‐layer Nb_2_C (Figure S1, Supporting Information), as evidenced by transmission electron microscopic (TEM) and atomic force microscopic (AFM) images (Figures S2 and S3, Supporting Information).^[^ ^14^ ^]^ Due to the large surface area and positive‐charged surface of Nb_2_C NSs, Au nanoparticles (NPs) with 20 nm in diameter (Figure S4, Supporting Information) are accessible to be integrated onto their surfaces via electrostatic interactions to yield Nb_2_C/Au, as evidenced by the presence of Au elements in Nb_2_C/Au NSs (Figure S5, Supporting Information). Furthermore, anti‐TNF*α* is also expected to reside on Nb_2_C NSs via electrostatic adsorption to obtain the Nb_2_C/Au/anti‐TNF*α* composite. It is found that Au NPs are uniformly‐distributed on Nb_2_C/Au/anti‐TNF*α* nanocomposite (Figure 1b,c), suggesting anti‐TNF*α* loading fails to alter distribution of Au NPs. As well, the presence of Au on Nb_2_C/Au/anti‐TNF*α* composite are verified again via energy disperse spectroscopy (EDS) and atom mapping (Figure 1d,e). Intriguingly, the presences of N and P in Nb_2_C/Au/anti‐TNF*α* composite also indicate the successful entrapment of anti‐TNF*α* (Figure 1e). As well, the newly‐emerging characteristic peaks at ≈1421 and ≈872 cm^−1^ further demonstrate anti‐TNF*α* grafting on Nb_2_C/Au/anti‐TNF*α* composite (Figure S6, Supporting Information). Notably, the sequential adhesions of Au NPs and anti‐TNF*α* trigger the increased surface potentials in sequence (Figure 1f), but fails to vary particle size (Figure 1g). Afterwards, polyvinyl pyrrolidone (PVP) modification was carried on to stabilize the nanocomposite and prevent anti‐TNF*α* leakage, and the evident shifts of characteristic peaks at 1636 and 872 cm^−1^ suggest the successful PVP modification (Figure S6, Supporting Information). PVP modification brings about the synchronous increments of surface zeta potential and particle size (Figure 1f,g). Inspiringly, Au NPs loading, anti‐TNG*α*, and PVP modification are disabled to weaken Nb_2_C crystallinity and vary the valences of Au and Nb (Figures S7 and S8, Supporting Information). In particular, benefiting from the hydrophilicity and electronegativity, Nb_2_C/Au/anti‐TNF*α*‐PVP displays favorable stability and dispersity in various physiological solutions (Figure 1h). Notably, once entering an acidic medium mimicking tumor microenvironment), PVP molecules are shed, causing the decreases of surface zeta potential and particle size (Figure S9, Supporting Information). The loading percentages of Au and anti‐TNF*α* in Nb_2_C/Au/anti‐TNF*α*‐PVP were determined to be 9.7% and 0.144% via inductively coupled plasma‐atomic emission spectrometry (ICP‐AES) method and UV–vis absorbance‐concentration standard curve test (Figure S10, Supporting Information), respectively.

### 2.2 In Vitro Phototherapy Efficiency against 4T1 Cells

**Figure 2:**
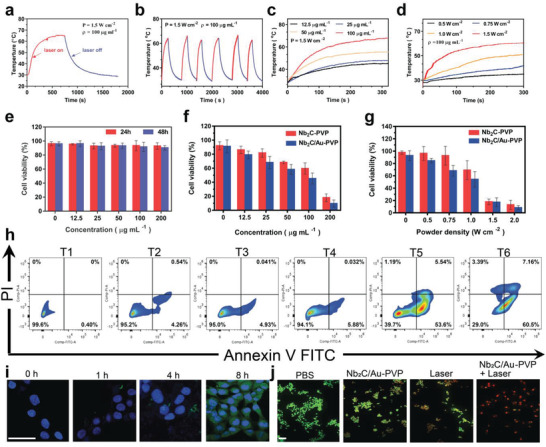
In vitro phototherapy survey including performance evaluation and anti‐tumor therapy. a) Time‐dependent photothermal curve of Nb_2_C/Au/anti‐TNF*α*‐PVP nanocomposite dispersion under NIR‐II laser irradiation, where laser irradiation would not cease until a steady‐state temperature was reached, followed by a cooling period after laser off; b) repeated heating curves of the dispersed Nb_2_C/Au/anti‐TNF*α*‐PVP suspension within six laser on/off cycles with certain intervals. c) Time‐dependent photothermal transformation curves of Nb_2_C/Au/anti‐TNF*α*‐PVP as a function of Nb concentration in the presence of 1064 nm laser irradiation (0.75 W cm^−2^). d) Time‐dependent photothermal transformation curves of Nb_2_C/Au/anti‐TNF*α*‐PVP as a function of power density at a fixed Nb concentration ([Nb] = 100 µg mL^−1^). e,f) Relative viabilities of 4T1 cells after incubation with PVP‐modified Nb_2_C or (both) PVP‐modified Nb_2_C/Au nanocomposites with varied Nb concentrations in the presence (e) and absence (f) of 1064 nm laser irradiation (0.75 W cm^−2^) for 24 and 48 h, respectively. g) Relative viabilities of 4T1 cells after incubation with PVP‐modified Nb_2_C and PVP‐modified Nb_2_C/Au in the presence of 1064 nm laser irradiation with varied power densities (0.75 W cm^−2^) for 24 and 48 h, respectively. h) FCM analysis on 4T1 cell apoptosis after different treatments (T1: PBS, T2: Nb_2_C/anti‐TNF*α*‐PVP, T3: Nb_2_C/Au/anti‐TNF*α*‐PVP, T4: laser alone, T5: Nb_2_C/anti‐TNF*α*‐PVP +laser, T6: Nb_2_C/Au/anti‐TNF*α*‐PVP+laser). i) LCSM images of 4T1 cells treated with FITC‐labeled Nb_2_C/Au/anti‐TNF*α*‐PVP for 0, 1, 4, and 8 h, respectively. Scale bar: 20 µm. j) LCSM images of 4T1 cells stained by calcein AM (green) and propidium iodide (PI, red) after different treatments. Scale bar: 100 µm. Laser parameters: wavelength‐1064 nm, power density‐0.75 W cm^−2^ and the dose: [Nb] = 100 µg mL^−1^. Data were expressed as mean ± SD (*n* = 8).

Akin to previous reports, Nb_2_C NSs are equipped with high photothermal conversion efficiency and photothermal stability within the NIR‐II window (1064 nm) (**Figure** **2**a,b), which determines that Nb_2_C NSs as the classic photothermal‐conversion materials are expected to enable the phototherapy of Nb_2_C/Au/anti‐TNF*α*‐PVP composite.^[^ ^14^ ^]^ To demonstrate it and screen the appropriate parameters, systematic experiments have been made. In light of the phenomenon that rapid precipitation occurs in Nb_2_C‐based NPs (i.e., Nb_2_C/Au/anti‐TNF*α*) without PVP within 1 h in comparison to PVP‐modified ones (Figure S11a, Supporting Information), NPs without PVP modification are inapplicable for in vitro performance evaluation and cellular‐level and in vivo experiments. Therefore, PVP‐modified ones were used in all experiments. The time‐dependent temperature variation profiles of Nb_2_C‐PVP are acquired, wherein a higher laser power density or/and larger Nb_2_C‐PVP concentration result in a considerably‐increased magnitude of temperature (Figure 2c,d). Consequently, higher cell deaths are observed under higher laser power density or/and larger Nb_2_C‐PVP concentration (Figure 2f,g), wherein the Nb_2_C‐PVP NSs exhibit neglectable cytotoxicity (Figure 2e). Intriguingly, chelated Au NPs also contribute to massive cell deaths in Nb_2_C/Au‐PVP due to Au NPs‐mediated heat conduction and photothermal conversion (Figure 2f,g). As well, the IC50 values of Nb_2_C‐PVP and Nb_2_C/Au‐PVP in concentration and laser power density were determined. It is found that Au chelation allow the IC50 values to drop from 1.166 to 1.096 W cm^−2^ in power density and from 94.07 to 56.48 µg mL^−1^ in Nb concentration (Figure S11b–e, Supporting Information).

Flow cytometry (FCM) analysis was used to verify aforementioned phototherapy‐based anti‐tumor results via CCK8 assay. Nb_2_C/Au/anti‐TNF*α*‐PVP will not kill cells, while once combining with NIR‐II laser (1064 nm) irradiations, it exerts the most robust killing effect (Figure 2h) because of the accumulative Nb_2_C/Au/anti‐TNF*α*‐PVP retention (Figure 2i) and Nb_2_C/Au/anti‐TNF*α*‐PVP photothermal‐induced temperature rise in tumor cells. Similar results are obtained via laser confocal scanning microscopy (LCSM) observation after calcein‐AM/propidium iodide (PI) co‐staining post‐treatments with different groups. Thanks to the massive accumulation of Nb_2_C/Au/anti‐TNF*α*‐PVP and the combined photothermal conversions of Nb_2_C and Au in Nb_2_C/Au/anti‐TNF*α*‐PVP, the red color representing dead cells almost illuminates the whole horizon in comparison to other groups (Figure 2j), which means that Nb_2_C/Au/anti‐TNF*α*‐PVP in the presence of NIR‐II laser irradiation induces the most apoptosis. Notably, the treatment in Nb_2_C/anti‐TNF*α*‐PVP+laser group also causes cell deaths via Nb_2_C photothermal‐induced temperature elevation, but the outcome is much inferior to Nb_2_C/Au/anti‐TNF*α*‐PVP +Laser.

### 2.3 In Vitro Antibacterial Assays

**Figure 3:**
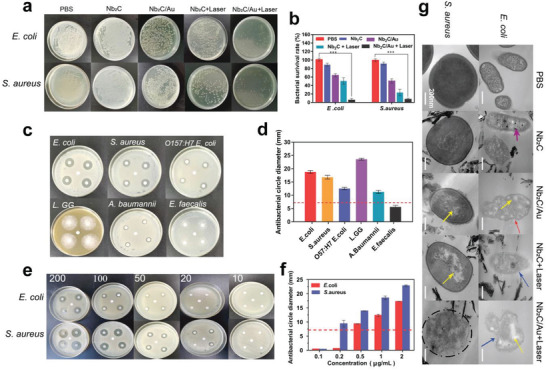
In vitro gut microbiome inhibition tests using such “chemical” and “physical” synergistic bactericidal effects consisting of Nb_2_C/Au‐PVP‐mediated phototherapy and inherent compositions. a,b) Digital photos (a) and quantitative statistical data (b) of *E. coli* (top panel) and *S. aureus* (bottom panel) bacterial in agar plates where *E. coli* and *S. aureus* bacterial were re‐cultivated after different treatments (PBS, Nb_2_C‐PVP, Nb_2_C/Au‐PVP, Nb_2_C‐PVP+Laser, and Nb_2_C/Au‐PVP+Laser) for 4 h. c,d) Bacteria‐inhibiting ring test including digital photo observation (c) and quantitative statistical data (d) where Nb_2_C/Au‐PVP treatment was used to inhibit different bacteria including *E. coli*, *S. aureus*, *O157:H7 E. coli*, *L. GG*, *A. baumannii*, and *E. faecalis*. e,f) Concentration‐dependent antibacterial activities of Nb_2_C/Au‐PVP in bacteriostatic ring test ([Nb] = 10, 20, 50, 100, and 200 µg mL ^−1^) including digital photo observation (e) and quantitative statistical data (f). Note, the diameter ≥ 7 mm of inhibition ring (d,f) was determined to be of antibacterial effect (red dotted line above). g) TEM images of *E. coli* and *S. aureus* that experienced different treatments, scale bar: 200 nm. Note, [Nb] = 100 µg mL^−1^, laser power density = 0.75 W cm^−2^. Statistical significance was determined by Student's *t*‐test at the univariate level, and ***p < 0.001. Data were expressed as mean ± SD (*n* = 3).

Inspired by above results, the excellent photothermal stability and transition efficiency of Nb_2_C/Au are also expected to decrease intratumoral microbiome abundance and paves a solid foundation to vary intratumoral gut microbiota metabolites and disrupt their metabolisms. These alterations of microbiome abundance, metabolism pathways, and metabolites are much preferable for further potentiating phototherapy‐mediated and tumor metabolism‐engineered anti‐cancer consequences since gut microbiome displayed positive correlations with tumor origin, progress, and metastasis.^[^ ^19^ ^]^ A general plate counting test was further harnessed to evaluate the antibacterial efficacy of Nb_2_C/Au‐PVP+Laser for *Escherichia coli* and *Staphylococcus aureus*. In both Nb_2_C‐PVP+Laser and Nb_2_C/Au‐PVP+Laser groups, significantly‐increased anti‐bacterial activities are observed, among which the Nb_2_C/Au‐PVP+Laser group shows the best antibacterial effect due to the highest photothermal conversion‐elevated temperature by both Nb_2_C and Au NPs (**Figure** **3**a,b). Besides phototherapy‐enhanced physical anti‐bacterial effect, Au and Nb_2_C also exert the robust “chemical” anti‐bacterial effect and result in an abundance drop of symbiotic microbiome, as evidenced by Nb_2_C‐PVP alone and Nb_2_C/Au‐PVP alone compared to control group. This “chemical” anti‐bacterial effect of Nb_2_C/Au‐PVP is attributed to the oxidation‐induced physical stress and mechanical damages to cell membranes.^[^ ^15^, ^20^ ^]^

To further evaluate the “chemical” and “physical” synergistic anti‐bacterial effects, bacteria‐inhibiting ring test on an agar culturing apparatus was carried out to explore the anti‐microbial activity of Nb_2_C/Au‐PVP against various symbiotic gut microbiomes including *E. coli, S. aureus, O57:H7 E. coli, L. GG, Acinetobacter baumannii*, and *Enterococcus faecalis*. Results show that the Nb_2_C/Au‐PVP is equipped with an excellent and wide antibacterial ability against most bacteria except *Enterococcus faecalis* (Figure 3c,d). Higher concentration can harvest more potent anti‐bacterial activities and higher inhibition rate objective to *E. coli* and *S. aureus* (Figure 3e,f).

Subsequently, bacteria morphology was evaluated to gain more insights into the synergistic bactericidal mechanism. In the PBS group, intact thick walls and cytoplasmic membranes are observed (Figure 3g). In treated groups (e.g., Nb_2_C‐PVP and Nb_2_C/Au‐PVP), the cytoplasmic structures of both *E. coli* and *S. aureus* are destroyed, and cytoplasmic components even outflow out of *E. coli* due to the more severe structure destruction. In the Nb_2_C‐PVP+Laser group, many air bubbles burgeon, resulting in the worsened morphology deformation and cytoplasmic structure destruction in both *E. coli* and *S. aureus*. Especially in Nb_2_C/Au‐PVP+Laser group, *S. aureus* membrane even disappears, leaving fuzzy cytoplasmic fragments, and *E. coli* morphology is severely distorted, featured of cytoplasm vacuolation and fragmentation (Figure 3g). These results indicate that the “chemical” and "physical synergistic bactericidal effects of Nb_2_C/Au‐PVP in the presence of laser irradiation can destroy the bacterial structure, enabling the modulations of abundance and metabolism of intratumoral commensal microbiota.

### 2.4 In Vivo Metabolism Modulations of Cancer and Gut Microbiota for Augmenting Phototherapy against 4T1 Breast Tumor

**Figure 4:**
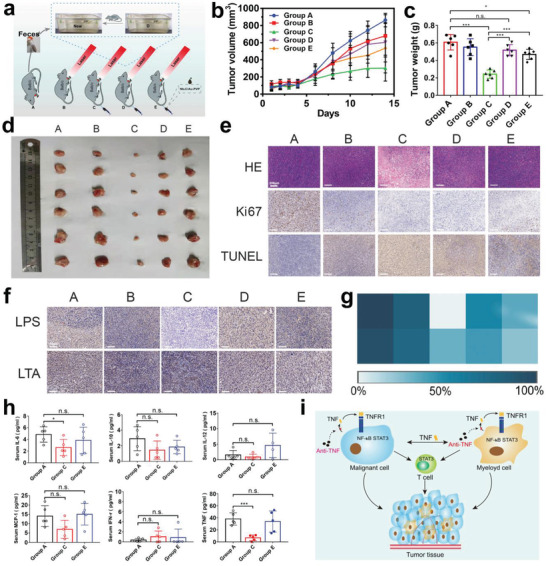
In vivo anti‐tumor explorations based on the abundance and metabolism disruption of intratumoral gut microbiome, tumor metabolism modulation, bacteria‐arisen inflammation inhibition as well as direct heat apoptosis in such Nb_2_C/Au/anti‐TNF*α*‐PVP‐gated phototherapy. a) Schematic on experimental grouping and detailed experimental procedures. b) Time‐dependent growth profiles of 4T1 tumors in different treatment groups; and c,d) tumor weight surveillance (c) and photographs (d) of excised 4T1 tumors at the end of experimental period. e) Optical microscopic images of harvested tumor slices after different corresponding treatments and several immunohistochemical stainings including hematoxylin and eosin (H&E) staining, terminal deoxynucleotidyl transferase dUTP nick end labeling (TUNEL) staining, and Ki67 staining in groups A‐E; scale bar: 200 µm. f) Consecutive sections of harvested tumor slices stained with anti‐LPS antibody (LPS), anti‐LTA antibody (LTA) after different treatments in Groups A–E; scale bar, 200 mm. g) Heat map for examining the proportion of tumor cells that were positively stained by LPS or LTA after different corresponding treatments in Groups A–E. h) The expression levels of serum IL‐ 6, IL‐10, IL‐12, MCP‐1, IFN‐*γ*, and TNF in Group A, Group C, and Group E using ELISA assay method. i) Mechanistic schematic depicting the pathways that entrapped anti‐TNF*α* mitigate bacterial‐incurred inflammation. Groups A–E represent PBS, Laser, Nb_2_C/Au/anti‐TNF*α*–PVP+Laser, Nb_2_C/Au/anti‐TNF*α*–PVP+Laser (cage change), and Nb_2_C/Au‐PVP+Laser, respectively. In Group D, the treatment method was identical to that of Group C, but after treatment, mice were transferred to the fecal environment of tumor‐bearing mice in Group A (Control). Laser parameters: wavelength‐1064 nm, power density‐0.75 W cm^−2^, duration‐6 min. Data were expressed as mean ± SD (*n* = 6). Student's and two‐tailed *t*‐test methods were used to indicate the significance, and n.s. – no significant; *p ˂ 0.05, ***p ˂ 0.001.

All in vivo experiments were performed according to protocols approved by the Laboratory Animal Center of Shanghai Tenth Peoples' Hospital (approval number: SHDSYY‐2020‐3429) and were in accordance with the policies of National Ministry of Health. It has been documented that intestinal microbiota can drive tumor to reorganize its metabolism and produce enough energy and biosynthetic components or metabolic intermediates such as nucleotides, lipids, amino acids, fumarate, and 2‐hydroxyglutarate for nourishing malignant cells, promoting their proliferation and tumorigenesis.^[^ ^7^ ^]^ Inspired by it, the abundance and metabolism disruptions of commensal microbiota will allow in vivo tumor repression via varying metabolism and metabolic pathways of tumor microenvironment on 4T1 tumor‐bearing mice (**Figure** **4**a). Herein, anti‐TNF*α* was added to ease bacterial inflammation and PVP modification was introduced to improve their stability. Before anti‐tumor evaluations, in vivo pharmacokinetics and bio‐distributions of Nb_2_C/Au/anti‐TNF*α*‐PVP were first explored. The long half‐life (i.e., 130.7 min) and high intratumoral accumulation are obtained (Figure S12, Supporting Information). As well, in vivo animal imaging and ex vivo tissue imaging also indicate the highly‐efficient retention of Nb_2_C/Au/anti‐TNF*α*‐PVP in tumor (Figure S13, Supporting Information). The inspiring high accumulation will sufficiently ensure the excellent anti‐tumor outcomes.

Time‐dependent variation profiles of tumor volume provide a clear scenario that the metabolism homeostasis disruptions of tumor and gut microbiota as well as their abundance alteration in induced mild phototherapy confer Nb_2_C/Au/anti‐TNF*α*‐PVP with the most potent ability to repress tumor growth in the presence of laser irradiation (Group C). However, once the treated mice return to and are kept in the fecal environment of control mice (Group A), the excreted gut microbiota metabolites in Group A will reignite or revoke the growth lust of tumor in Group D. On this account, the treatment outcomes including tumor volume and weight in Group D are inferior to those in Group C (Figure 4b–d and Figure S14, Supporting Information). The therapeutic efficacy in vivo was evaluated by various pathological examinations. It is found that the Nb_2_C/Au/anti‐TNF*α*‐PVP+Laser group induces the most nuclei damages represented by intranuclear debris and thus brings about the most cell deaths, accompanied with the considerably‐suppressed tumor cell proliferation as evidenced in Figure 4e. TUNEL staining denotes that the largest degree of apoptosis pathway activation is responsible for the most cell deaths in the Nb_2_C/Au/anti‐TNF*α*‐PVP+Laser group (Figure 4e).

To understand the anti‐tumor principle, immunohistochemistry (IHC) staining using antibodies against bacterial lipopolysaccharide (LPS) and lipoteichoic acid (LTA) was carried out to detect Gram‐negative and Gram‐positive bacteria in mammary 4T1 tumors that experienced different treatments. Bacterial LPS and LTA are detected in all subgroups of tumors featuring uniform spatial distribution (Figure 4f,g), but the abundance of bacteria in Group C significantly drops. Concurrently, the inflammatory cytokine levels (e.g., IL‐6, TNF‐*α*) in mice serum in Group C, are tremendously down‐regulated due to the presence of anti‐TNF*α* in comparison to other two groups without anti‐TNF*α* (Figure 4h). This intriguing phenomenon demonstrates that anti‐TNF*α* loading also contributed to the considerably‐delayed tumor progression in Group C via activating the anti‐inflammation signal pathways and down‐regulating the pro‐inflammatory factors (e.g., IL‐6 and TNF‐*α*) (Figure 4i) since inflammation also closely correlate with tumor origin and progression.^[^ ^17^, ^21^ ^]^ The progression of tumor was delayed by regulating the transitional expression of inflammatory factors within the tumor. In this singling axis, T cells were regulated, and the Nb_2_C/Au/anti‐TNF*α*‐PVP+Laser group (Group 3) brings about the most CD8+ T infiltrations for anti‐tumor immunotherapy and reduce CD4+ T level (Figure S15, Supporting Information).

Above all results adequately shed light on the anti‐tumor principles. In detail, the composition and phototherapy deriving from Nb_2_C/Au/anti‐TNF*α*‐PVP in the presence of laser irradiation could bring about the “chemical” and "physical synergistic bactericidal effects. Afterwards, they disrupt intestinal microbiome ambulance/metabolites, alleviate intratumoral symbiotic bacteria‐arisen inflammation, and concurrently tailor tumor cell metabolites (Scheme 1). These intratumoral metabolisms and inflammation variations of microbiota and tumor cells could further induce necrosis, inhibit tumor cell proliferation, and unfreeze tumor treatment resistance, thereby enabling the magnification efficiency of commensal microbial modulation‐mediated anti‐tumor. Additionally, routine blood, blood biochemistry, and hematoxylin‐eosin (H&E) immunochemical staining images show no significant differences between groups, indicating excellent tolerance of Nb_2_C/Au/anti‐TNF*α*‐PVP, which ensures in vivo applicability (Figures S16 and S17, Supporting Information). In addition, no significant decrease in body weight also indicates the excellent biocompatibility (Figure S18, Supporting Information).

### 2.5 Intratumoral Microbiome Metabolism Disruption Analysis

**Figure 5:**
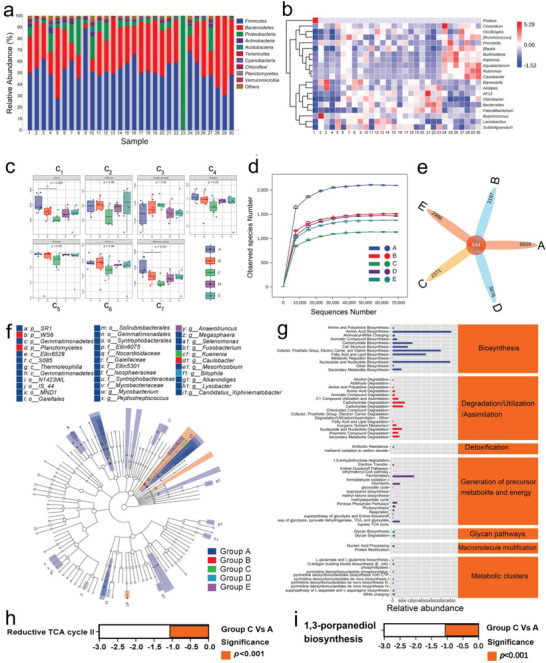
Flora analysis in 4T1 tumor for evaluating the metabolism disruption of intratumoral intestinal microbiome by the phototherapy. a) Bacterial taxonomic profiling of tumor microbiota at phylum level; and b) heat map of tumor bacteria based on of top 20 genera in relative abundance; c) abundance and alpha diversity of microbial in 4T1 tumor. Note, Chao1 (c_1_) and Observed species (c_7_) indices serve to characterize microbiome richness; Shannon (c_4_) and Simpson (c_5_) indices reflect diversity; Faith's PD index (c_2_), Pielou's evenness index (c_3_), and Good's coverage index (c_6_) were used to assess the evolutionary‐based diversity, evenness and coverage. Values are means ± SD (*n* = 6). Student's *t*‐test was used to indicate the significance, and *P < 0.05, and evident difference with statistical significance between Groups A and C is observed according to Chao1 (c_1_) and Observed species (c_7_) indices. d) Rarefaction curves for expressing the richness of sample species in different groups; e) Wayne diagram of OTUs to analyze the diversity of bacterial species among different treatment groups. f) LEfse (linear discriminant analysis and effect size) dendrogram, wherein LEfSe was used to analyze the classification tree of sample species and find the marker species (i.e., metabolites) with significant differences in different groups; g) abundance statistics diagram of bacterial metabolic pathways; h,i) analysis of differential metabolic pathways between Group A and Group C in terms of reductive TCA cycle (h) and 1,3‐porpanediol biosynthesis (i). Note: Groups A–E represent Control, Laser alone, Nb_2_C/Au/anti‐TNF*α*‐PVP+Laser, Nb_2_C/Au/anti‐TNF*α*‐PVP+Laser (cage change), and Nb_2_C/Au/‐PVP+Laser, respectively, and in Group D, the treatment method was identical to that of Group C, but after treatment, mice were transferred to the fecal environment of tumor‐bearing mice in Group A (Control). Laser parameters: wavelength‐1064 nm, power density‐0.75 W cm^−2^, duration‐6 min.

Although the relationship between bacteria, inflammation, and tumors has been more widely accepted and understood, the singling pathways and immune metabolic mechanisms remains unclear, and there is few researches on how metabolites affects tumor development. Fortunately, the precise modulations of bacterial abundance and metabolism furnish an ideal tool to comprehensively understand the ecology that affects tumor. To recognize and figure out the roles of metabolism modulations associated with intratumoral microbiota and tumor microenvironment, 16S rDNA gene sequencing technology was adopted to analyze their variations of abundance and metabolite. After taxonomic composition analysis, the main bacterial composition in each sample was identified. No significant difference in the diversity and dispersion is observed (**Figure** **5**a,b), suggesting the reliability of 4T1 xenografted tumor for exploring the feasibility of microbiota metabolism and inflammation modulations‐based anti‐tumor therapy. It is found that the relative abundance in treatment groups (C and D) is much lower than that in other groups (Figure 5c,d), and Group C harvests the lowest richness of intratumoral microbiota. Notably, the mice in Group D were transferred and fed in cages containing the mouse feces harvested from the control group after Nb_2_C/Au‐TNF*α*‐PVP+Laser treatment, consequently elevating the relative abundance of flora due to the recharge of gut microbiome in feces into tumors in comparison to Group C (Figure 5c,d). Regarding this, the bacteria in the tumor may be affected by the surrounding breeding environment. To further understand it, bacteria type was monitored, and the proprietary bacteria type in Group C (2371) is much lower than that in other groups including Group D (3876) in the petal/Venn diagram (Figure 5e). As well, the marked microbial taxa that behaved consistently in different subgroups were further studied via LEfSe analysis, and the marked species with high abundance are mostly present in control group, which is much more than that in treatment groups (Figure 5f). These results suggest that these screened organisms may serve as the tumorigenic factors to resist tumoricidal therapy, which is probably mediated by secreting metabolites to directly promote tumor growth or indirectly affect tumor metabolism microenvironment for improving the tolerance.

To further understand it, the metabolites and major metabolic pathways of intratumoral microbiome and tumor cells were monitored as well as bacteriophage functions and metabolic delivery abundance. According to 7.1 (PICRUSt2) analysis (Figure 5g), various metabolic pathways within the major classes (e.g., biosynthesis, degradation/utilization/assimilation, detoxification, generation of precursor metabolite and energy, glycan pathways, macromolecule modification, and metabolic clusters) are obtained. Further analysis of metabolic pathway differences between groups was carried out. In comparison to control group, several metabolism pathways of intratumoral microbiota in Nb_2_C/Au/anti‐TNF*α*‐PVP+Laser group are varied, among which the metabolic pathways, that is, reductive TCA cycle II and 1,3‐porpanediol biosynthesis, exhibit the significant differences (*p* < 0.001) (Figure 5h,i). On this account, the treatment in Group C may down‐regulate products such as lactic acid, thereby contributing to the suppressed occurrence and development of tumors.

### 2.6 Quality Control in Tumor Microenvironment Metabolism Disruption Exploration

Besides gut microbiota metabolism modulation, tumor microenvironment metabolism can be concurrently altered by direct phototherapy and commensal bacterial metabolite alteration. Before evaluating it, quality control (QC) involving instrument stability, experiment reproducibility, and data quality reliability was enforced. Results show that the instrument error‐arisen variation is trivial during the whole experiment (Figure S19, Supporting Information). A good correlation with the correlation coefficient > 0.9 is obtained in Perform Pearson correlation analysis of QC samples, indicating the high experimental repeatability (Figure S20, Supporting Information). The multivariate control charts of QC samples show the good reproducibility (Figure S21a,b, Supporting Information) and high equipment stability available for subsequent analysis (Figure S21c,d, Supporting Information). Overall, the instrumental analysis system is stable and the test data are stable and reliable. The differences in metabolic profiles obtained in the following tests can truthfully reflect the biological differences between these samples themselves.

To explore the alterations in 4T1 breast tumor mice and to investigate the probable treatment mechanism of the Nb_2_C/Au/anti‐TNF*α*‐PVP+Laser, further analysis of primary metabolite components was carried out, the significant separation of clusters among the groups of treated with or without fecal microbiota and control was evidenced by orthogonal projections to latent structures‐discriminate analysis (OPLS‐DA) score plot (Figure S22a–f, Supporting Information) and permutation test plot of OPLS‐DA derived from the GC‐TOF/MS metabolite profiles of tumor (Figure S22g–l, Supporting Information).

All metabolites identified in positive and negative ion patterns are classified and their proportions were counted according to their chemical taxonomy (Figure S23, Supporting Information). In particular, differential metabolites with FC>1.5 or FC<0.67 and *p* < 0.05, are provided in volcano plots (Figure S24, Supporting Information), and these differential metabolites with up‐ or down‐regulations between different subgroups exert potent effects on tumor metabolism. The values of variable importance for the projection (VIP) and relative standard deviation (RSD) of each metabolism characteristic were also calculated, wherein OPLS‐DA VIP>1 and *p* < 0.05 in metabolomics were determined as the screening criteria for selecting differential metabolites as potential biomarkers. Additionally, relative standard deviation (RSD) threshold (20%) in all metabolic characteristics of QC samples was as set to maintain stability in this study.

### 2.7 Metabolic Pathway Analysis in Tumor Microenvironment Metabolism Disruption Exploration

**Figure 6:**
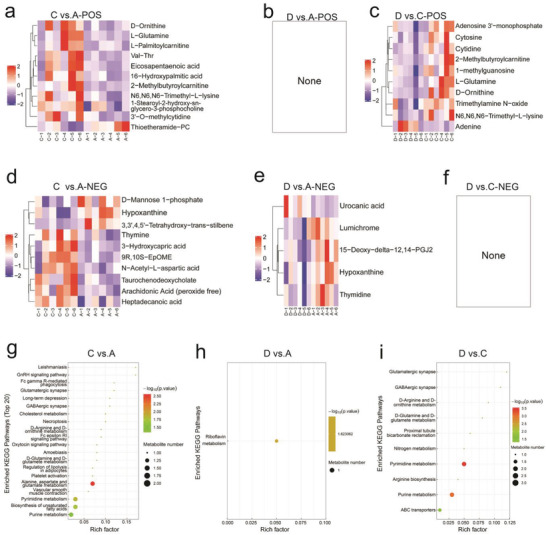
Metabolomics analysis based on UPLC‐QTOF/MS for uncovering tumor microenvironment metabolism disruption by the phototherapy. a–f) Heat maps of all differential metabolite clusterings in both POS (a–c) and NEG (d–f) ion modes between groups C and A (a,d), D and A (b,e), and D and C (c,f), respectively. Each row in the clustering heat map denotes a differential metabolite (i.e., the vertical axis indicates metabolites with significant differential expressions), while each column represents a set of samples (i.e., the horizontal axis shows the sample information). The red color represents significant up‐regulation, blue color represents significant down‐regulation, and the color shade indicates the degree of up‐ or down‐regulation. The metabolites with similar expression patterns are clustered under the identical cluster on the left. g–i) The bubble diagrams of KEGG enrichment pathways between groups C and A (g), D and A (h), and D and C (i), respectively. Note: Groups A–D represent Control, Laser alone, Nb_2_C/Au/anti‐TNF*α*‐PVP+Laser, and Nb_2_C/Au/anti‐TNF*α*‐PVP+Laser (cage change), respectively, and in Group D, the treatment method was identical to that of Group C, but after treatment, mice were transferred to the fecal environment of tumor‐bearing mice in Group A (Control). Laser irradiation parameters: wavelength‐1064 nm, power density‐0.75 W cm^−2^, duration‐6 min.

After above precise and reliable QC, mass spectrometry (MS) fragments in different groups were recognized and compared within a secondary commercial database to match metabolism characteristics (Tables S1–S3, Supporting Information). Results show that Group C treatment receives 21 differential metabolites compared with Group A (**Figure** **6**a,d and Table S1, Supporting Information). Intriguingly, fewer differential metabolites between D and A Groups are observed, for example, 0 and 5 in POS ad NEG ion patterns, respectively. This phenomenon indicates that the mice feces in Group A that are placed in Group D cage re‐arrange the treated mice in Group D in the gut microbiome‐enriched environment, which favors to re‐supply microbiome into tumor and enable the re‐activation of normal tumor growth and metabolism in Group D (Figure 6b,e and Table S2, Supporting Information). In this regard, contributed by the re‐charge of microbiota, the number of differential metabolites in Group D is 10 compared with Group C (Figure 6c,f and Table S3, Supporting Information).

The KEGG enrichment analysis was carried out through Fisher's Exact Test to analyze and calculate the significance level of each metabolite enrichment pathway to identify abnormal metabolism and varied signal transduction pathways. Results reveal that 20 significantly‐different metabolic pathways between Groups A and C are found (Figure 6g), corresponding to above 20 differential metabolites (Figure 6a,d). However, in the number between Groups D and A is only one because of the gut microbiota re‐charge in flora microenvironment by mouse feces in control group (Figure 6h), which consequently impairs the therapeutic outcomes (Figure 4b–d). Corresponding to the number of differential metabolites, there are 10 differential tumor metabolism pathways between Groups D and C (Figure 6i), which can be attributed to the recovered flora surrounding in Group D. In addition, metabolic proximities between significantly different metabolites (i.e., VIP > 1, *p* < 0.05) in both positive and negative correlation analyses were inspected since they are synergistically interconnected or mutually exclusive with each other (Figure S25, Supporting Information), wherein the metabolic proximities of differential metabolites between Group C and Groups A or D are poor, suggesting the occurrence of metabolism disruption‐arisen function variation in Group C. These results adequately demonstrate that this intratumoral microbiota metabolism‐engineered anti‐tumor treatment exerted significant impacts on tumor metabolisms via direct phototherapy and microbial metabolism disruption mediation, which, along with anti‐inflammation and photothermal ablation, were responsible for magnifying aforementioned tumor treatment consequences.

### 2.8 Generality Validation of Such Nanocomposites on CT26 Tumor Model

**Figure 7:**
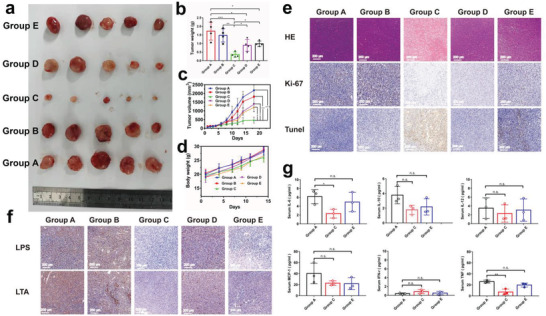
In vivo anti‐tumor evaluations using Nb_2_C/Au/anti‐TNF*α*‐PVP on CT26 tumor model to verify the generality. a,b) Digital photos (a) and weights (b) of harvested tumors from CT26 tumor‐bearing mice that experienced corresponding treatments in Groups A–E at the end of experimental period. c,d) Time‐dependent tumor growth profiles (c) and body weights (d) of CT26 tumor‐bearing mice model after experienced corresponding treatments in Groups A‐E. e) Optical microscopic images of harvested tumor slices after different corresponding treatments and several immunohistochemical stainings including H&E, TUNEL, and Ki67 in Groups A–E; scale bar: 200 µm. f) Consecutive sections of harvested tumor slices stained with anti‐LPS and anti‐LTA after different treatments in Groups A–E; scale bar, 200 mm. g) The expression levels of serum IL‐ 6, IL‐10, IL‐12, MCP‐1, IFN‐*γ*, and TNF*α* in Groups A, C, and E using ELISA assay method. Data were expressed as mean ± SD (*n* = 5). Student's and two‐tailed *t*‐test methods were used to indicate the significance, and n.s. – no significant; *p ˂ 0.05, **p ˂ 0.01, and ***p ˂ 0.001.

To validate the abundance and metabolism disruptions of intratumoral microbiota by such chemical and physical actions for repressing tumor, another tumor model (i.e., CT26) was used. Identical results were acquired. In detail, the most retention of Nb_2_C/Au/anti‐TNF*α*‐PVP in CT 26 tumor is found (Figure S26, Supporting Information), ensuring the excellent anti‐tumor outcomes. Nb_2_C/Au/anti‐TNF*α*‐PVP+Laser (Group C) significantly represses tumor growth (**Figure** **7**a–c) without altering body weights (Figure 7d) via inducing the most tumor apoptosis and inhibiting cell expansion (Figure 7e). Gram‐negative and Gram‐positive bacteria in CT26 tumors that experienced differently were monitored via IHC staining using antibodies against LPS and LTA. Akin to above results on 4T1 tumor model, Nb_2_C/Au/anti‐TNF*α*‐PVP+Laser treatment harvested the most significantly‐decreased abundance of bacteria (Figure 7f). Concurrently, the inflammatory cytokine levels (e.g., IL‐6, TNF‐*α*) in mice serum in Group C, are tremendously down‐regulated due to the presence of anti‐TNF*α* in comparison to other two groups without anti‐TNF*α* (Figure 7g). These results validate that the abundance and metabolism disruptions of intratumoral microbiota by such chemical and physical actions in such nanocomposites can serve as a general method to treat other tumors.

## 3 Conclusions

In summary, we engineered an intratumoral microbiota metabolism‐engineered nanocomposite (Nb_2_C/Au/anti‐TNF*α*‐PVP) for regulating the abundance and diversity of a commensal microorganism and disrupting the metabolic pathways of intratumoral microbiota and tumor microenvironment associated with the types and levels of metabolites and biosynthetic substances that tumor growth demanded. In vitro experiments demonstrated that the compositions (i.e., Nb_2_C and Au) and phototherapy via photothermal conversion in the presence of NIR‐II laser irradiation allow “chemical” and “physical” anti‐bacterial actions, thus endowing the nanocomposite with robust antibacterial activity. More significantly, flora analysis and mass spectrometry (MS) and metabonomics combined analyses demonstrate that the “chemical” and “physical” bacterial regulation could decrease the abundance and diversity of intratumoral symbiotic microbiota, producing multiple differential metabolites, and change multiple metabolic pathways that could lead to tumor cell apoptosis. These systematic tests indicated the regulation of symbiotic flora played an important role in tumorigenesis and treatment, answering what and how microbiota metabolism and singling pathway manipulate tumor progression and uncovering the underlying principles. In addition, the plasticity of microbiota that could slow cancer progression by targeting inflammation was altered by introducing anti‐TNF*α*. The excessive inflammatory factors in the tumors were down‐regulated, which united with the “chemical” and “physical” bacterial regulation to synergistically augment phototherapy‐based anti‐tumor therapeutic consequences. This metabolism regulation strategy of intratumoral microbiota and tumor microenvironment provides us a new anti‐tumor means, and the underlying principles can serve as a general method against 4T1 and CT26 tumors to enlighten different communities to develop more microbiota regulation‐associated therapeutic agents against diverse lesions.

## Conflict of Interest

The authors declare no conflict of interest.

## Author Contributions

F.K., C.F., and Y.Z. contributed equally to this work. K.Z. conceived this project, and K.Z. and F.K. designed the project. F.K., C.F., Y.Z., L.D., D.D., G.X., X.L., H.L., and Y.Y. performed the experiments. K.Z. and F.K. analyzed the data and wrote the manuscript, and K.Z. revised the manuscript. H.X. supervised the project. H.X. and K.Z. commented on this manuscript.

## Supporting information

Supporting Information

## Data Availability

The data that support the findings of this study are available from the corresponding author upon reasonable request.
