# Supplementary material for: Abundance and Metabolism Disruptions of Intratumoral Microbiota by Chemical and Physical Actions Unfreeze Tumor Treatment Resistance
Source: Adv Sci (Weinh). 2022 Jan 17;9(7):2105523. doi: 10.1002/advs.202105523 (PMC8895135; doi:10.1002/advs.202105523)
Supplement: Supplementary file 1 — Supporting Information [file ADVS-9-2105523-s001.pdf]

ADVANCED SCIENCE
Open Access

Supporting Information

for *Adv. Sci.,* DOI: 10.1002/advs.202105523

Abundance and Metabolism Disruptions of Intratumoral Microbiota by Chemical and Physical Actions Unfreeze Tumor Treatment Resistance

*Fanlei Kong, Chao Fang, Yan Zhang, Lixia Duan, Dou Du, Guang Xu, Xiaolong Li, Hongyan Li, Yifei Yin, Huixiong Xu, and Kun Zhang**

Supporting Information for

Abundance & Metabolism Disruptions of Intratumoral Microbiota by Chemical and Physical Actions Unfreeze Tumor Treatment Resistance

Part A: ***Experimental Section***

Part B: ***Supplementary Tables***

Part C: ***Supplementary Figures***

Part A: *Experimental Sections*

1. Materials.

Nb_2AlC powders were purchased from the Forsman Scientific Co., Ltd. Hydrofluoric acid (HF, 49 wt%), tetrapropylammonium hydroxide (TPAOH) aqueous solution and N,N-Dimethylformamide (AR grade, DMF) were ordered from J&K Scientific Co., Ltd. Au (anatase) and polyvinyl pyrrolidone (PVP, MW = 40000) were purchased from Millipore Sigma Co., Ltd. Tetrapropylammonium hydroxide (TPAOH), Sodium borohydride ($NaBH_4$), Chloroauric acid ($HAuCl_4$), Ammonium acetate (NH_4AC), ammonium hydroxide (NH_4OH), ammonium fluoride (NH_4F), and formic acid (FA) were purchased from Sigma-Aldrich.. Roswell park memorial institute (RPMI) 1640 medium, penicillin-streptomycin, dulbecco's modified eagle medium (DMEM), fetal bovine serum (FBS). Anti-TNFα antibody [52B83], LTA4H polyclonal antibody, LPS antibody were purchased from abcam Co., Ltd. CCK8 assay kit and DAPI assay kit were obtained from Beyotime Biotechnology Co., Ltd. Annexin V, FITC Apoptosis Detection kit was purchased from Dojindo Laboratories. MS Inflammation CBA kit was obtained from BD Co., Ltd.

2. Characterizations.

The structure and morphology of different samples were characterized on Transmission electron microscopy (TEM, JEM-2100F), scanning electron microscopy (SEM, Magellan XHR 400) and atomic force microscopy (AFM Dimension Icon), respectively. X-ray photoelectron spectroscopy (XPS, ESCAlab250), Element mapping (Magellan 400), X ray diffraction (XRD, Bruker D8), Raman microscopy (Renishaw inVia, excitation wavelength: 532 nm) and Infrared spectrometer (FT-IR, Nicolet IS10) were used to analyze the element, composition and valence of these samples. Particle size and zeta potential were measured on Zetasizer Nanoseries (Nano ZS90). Absorption

spectra were obtained by Ultraviolet–visible spectroscopy (Uv-vis, UV-3600, Shimadzu). The samples were quantified *via* the inductively coupled plasma-optical emission spectrometry (ICP-OES, Agilent 725). The laser confocal scanning microscopy (LCSM) observation was implemented on FV1000 (Olympus Company). Flow cytometry data was obtained on BD FACS Aria II equipped with BD FACS Diva™ software.

3. Nb_2C nanosheets synthesis

Ultrathin Nb_2C nanosheets were synthesized according to a previous protocol. Briefly, Nb_2AlC (2 g) was added into hydrofluoric acid (50 mL, 49 wt %) under magnetic stirring at room temperature for two days. After centrifugation and washing with deionized (DI) water three times, the mixture was re-dispersed in TPAOH aqueous solution (50 mL) under magnetic stirring for another three days at room temperature. The intercalated Nb_2C nanosheets were obtained by centrifugation (11,000 rpm, 15 min) and then washed with ethanol and DI water to remove the redundant solvent.

4. Au NPs synthesis.

Au nanoparticles were synthesized according to a classic protocol. Briefly, 40 mL of $HAuCl_4$ (1.42 mM) was mixed with 400 μL of cysteamine solution (213 mM) and stirred for 20 min at room temperature. Subsequently, 10 μL of $NaBH_4$ solution (10 mM) was added and the mixture was kept in the darkness for 10 min at room temperature. After further stirring for 30 min, the resulting solution was stored at 4 °C.

5. Au NPs chelation on Nb_2C NSs.

Above synthesized Nb_2C NSs dispersion (5 mL) was mixed with the prepared growth solution (50 mL), followed by adding 5 mL of Au NPs aqueous solution (1 mg/mL) and 1 mL of formaldehyde, respectively. After sonication under room temperature for 4 h，the mixture was transferred into an

autoclave and placed in an oven for 12 h at 60 °C. Subsequently, Nb_2C/Au nanocomposites were collected by centrifugation (11,000 rpm, 20 min) and washed with DI water three times.

6. Surface PVPylation and anti-TNFα loading on Nb_2C/Au composites.

To stabilize and disperse Nb_2C/Au complexes, Nb_2C/Au complexes (10 mg) and polyvinylpyrrolidone (PVP) (50 mg) were added and dissolved in absolute ethanol, followed by constant stirring in a water bath at 50 °C for 6 h. Afterwards, the mixture were collected by centrifugation and washed with DI water three times. Subsequently, anti-TNFα (1 mL) was added into above solution and stirred for 24 h in the darkness. Eventually, the mixtures were collected by centrifugation and washed with DI water three times to obtain the final Nb_2C/Au/anti-TNFα-PVP aqueous solution, and the products were filled with argon against oxidation for ten minutes.

7. *In vitro* photothermal performance of Nb_2C/Au/anti-TNFα-PVP.

Infrared thermography was applied to record the temperature changes during laser irradiation so as to determine the *in vitro* photothermal properties of Nb_2C/Au/anti-TNFα-PVP. Temperatures in Nb_2C/Au/anti-TNFα-PVP solutions with different concentrations ([Nb] = 12.5, 25, 50, 100 μg mL^{-1}) were tested within 10 min in the presence of 1064nm NIR-II laser irradiation with different power densities (0.5, 0.75, 1.0, 1.25, 1.5 W cm^{-2}). Then, the photothermal stability of the Nb_2C/Au/anti-TNFα-PVP composite dispersion ([Nb] = 100 mg mL^{-1}) was determined under NIR-II laser irradiation (power density: 1.5 W cm^{-2}). In detail, when the steady-state temperature was attained, the laser was turned off and cooled for a period of time. Subsequently, the temperature changes of the Nb_2C/Au/anti-TNFα-PVP solution ([Nb] = 100 μg mL^{-1}) through six laser on/off cycles (1.5 W cm^{-2}) were recorded.

8. *In vitro* cell experiments.

4T1 (mouse breast cancer cell line) cells were incubated in roswell park memorial institute (RPMI) 1640 medium, wherein 10% fetal bovine serum and 1% penicillin/streptomycin were added. Cells were incubated in a CO_2 regulated incubator with an inhumidified 95% air/5% CO_2 atmosphere.

For *in vitro* cytotoxicity assay of Nb_2C/Au/anti-TNFα-PVP, 4T1 cells were seeded in 96-well plates at a density of 1×10^4 cells/well for 24 h to allow cell adhesion. Nb_2C/Au/anti-TNFα-PVP with different concentrations ([Nb]=0, 12.5, 25, 50, 100 and 200 μg mL^{-1}) were added into the cells and co-incubated for 24 h and 48 h, respectively, and subsequently their viability was determined using CCK-8 (Cell Counting Kit, Shanghai Beyotime Institute of Biotechnology, China). IC50 values were also surveyed by CCK8 method *via* tuning Nb concentrations or power densities.

9. *In vitro* synergistic therapy effect against 4T1 cells.

4T1 cells were seeded in 96-well plates at a density of 1×10^4 cells/well for 24 h to attach on the plates, and then co-incubated with Nb_2C-PVP and Nb_2C/Au-PVP at varied concentrations ([Nb] = 0, 12.5, 25, 50, 100, 200 μg mL^{-1}) for 6 h. Afterwards, these cells were exposed to 1064 nm laser irradiation for 5 min at 1.0 W cm^{-2}. In addition, to evaluate the cell viability after irradiations at different power densities (0, 0.5, 0.75, 1.0, 1.5, and 2.0 W cm^{-2}), 4T1 cells were co-incubated with Nb_2C-PVP and Nb_2C/Au-PVP at the same concentrations (100 μg mL^{-1}) for 30 min, and then these cells were irradiated for 5 min using 1064 nm laser with different power densities. Finally, a standard CCK-8 protocol was used to determine cell viabilities.

10. *In vitro* CLSM and flow cytometry observation of synergistic therapies effect of Nb_2C/Au&anti-TNFα-PVP.

LCSM observation and flow cytometry (FCM) analysis were used to qualitatively and quantitatively track intracellular endocytosis of Nb_2C/Au/anti-TNFα-PVP, respectively. In detail, 4T1 cells were

seeded in confocal-specific dishes and cultured overnight. After cell adherence, FITC-labeled Nb_2C/Au/anti-TNFα-PVP (100 μL) was added and incubated for different time periods (0 h, 1 h, 4 h, 8 h). After that, cell nuclei were stained by DAPI before LCSM observation. Then, 4T1 cells were co-incubated with PBS, Nb_2C/Au-PVP, laser and laser+Nb_2C/Au-PVP ([Nb] = 100 μg mL^{-1}) for 4 h, after which laser and laser+Nb_2C/Au group cells were irradiated for 5 min using 1064 nm laser at power densities 1.0 W cm^{-2}). After these treatments, 4T1 cells were observed by LCSM, wherein live cells and dead cells were stained by calcein-AM and PI, respectively.

FCM analysis was used to quantitatively assess cell apoptosis. Typically, 4T1 cells were seeded in 6-well plates for 24 h to allow them to attach on the dishes, and then the media was replaced by PBS, Nb_2C/Au-PVP and Nb_2C-PVP ([Nb] = 100 μg mL^{-1}) and incubated for 8 h. These cells were irradiated for 5 min using 1064 nm laser at different power densities (0, 1.0 W cm^{-2}). Afterwards, the cells were collected after a series of procedures including dissociation *via* trypsin, centrifugation and washing for 3 times with PBS. Finally, a mixture solution containing 5 μL PI and 5 μL FITC was added into these cells and incubated for 20 min, and immediately afterwards, FCM analysis was employed to evaluate the apoptosis level.

11. *In vitro* evaluation of anti-bacterial efficacy

Escherichia coli (*E. coli,* ATCC25922), *Staphylococcus aureus* (*S.aureus*, ATCC6538), *Escherichia coli O157:H7* (*E. coli O157:H7,* ATCC43888*), Lactobacillus rhamnosus GG* (*L.GG,* ATCC7469*), Acinetobacter baumannii* (*A.baumannii,* ATCC19606)*, Enterococcus faecalis* (*E.faecalis,* ATCC 29212) were obtained from China Shanghai Harmony Cell Technology Co., Ltd. All bacteria were freshly harvested from the bacterial culture after incubation at 37 °C for 12 h. *E. coli* and *A. baumannii* were cultured in LB broth (10 μg/mL, 10 mL). *S. aureus*, *E. faecalis*, *E. coli* O157:H7,

L.GG were cultured in the mixture of TSB (30 μg/mL, 10 mL) and yeast extract (5 μg/mL, 10 mL) (TSBY) broth. The resulting bacterial samples were centrifuged using a thermo centrifuge (model D-37520; Waltham, MA, USA) equipped with a rotor (radius = 8.0 cm) at 6000 rpm for 10 min, and the supernatant was removed. The collected bacterial cells were rinsed with PBS solution (0.15 M, 1 mL × 2) through centrifugation at 6000 rpm for 10 min. The obtained bacterial cells were re-suspended in PBS solution (1 mL, 0.15 M, pH 7.4). The antibacterial effect of different nanomaterials was verified by the inhibition ring test method on the six bacterial samples mentioned above. The test bacteria were inoculated on the surface of agar medium plate, and then the anti-bacterial samples were affixed, and the continuously-dissolved inhibition samples could be diffused into the agar and the concentration gradually decreased, giving birth to an inhibition ring. Therefore, the antibacterial ability and sensitivity were evaluated by measuring the width of inhibition ring. Similarly, Nb_2C/Au-PVP (0.5 mL) with varied concentrations (2 mg mL^{-1}, 1 mg mL^{-1}, 0.5 mg mL^{-1}, 0.2 mg mL^{-1} and 0.1 mg mL^{-1}) were added into the prepared *S. aureus* and *E. faecalis* bacteria solution, respectively. According to the inhibition ring test method, the width of inhibition ring was detected after 3 h.

E. faecalis and *S. aureus* suspensions (200 μL, 2×10^5 CFU mL^{-1}) were incubated with PBS, Nb_2C-PVP, Nb_2C/Au-PVP, Nb_2C-PVP +Laser, Nb_2C/Au-PVP+Laser (500 μg mL^{-1}) in 96-well plates, wherein the plates were irradiated with or without 1064 nm laser (1 W cm^{-2}) for 10 min according to the grouping. After the corresponding treatments, the plates were incubated at 37 °C for 12 h. Subsequently, the plates were gently shaken and the solution was diluted into 20-fold volume with PBS. 50 μL of bacteria solution was spread on LB plates and incubated at 37 °C for 24 h. Then the colonies on the plates were measured.

12. Bacteria morphology evaluation.

Bacterial morphology was observed using transmission electron microscopy (TEM). The *E.coli* bacteria and *S.aureus* bacteria treated with Nb_2C/Au-PVP with/without 1064 nm laser irradiation were fixed with 1% glutaraldehyde for 2 h at 4 °C, and then fixed at 30%, 50%, 70%, and 80% respectively. Subsequently, they were dehydrated in 90% and 100% fixed ethanol for 15 minutes, respectively, and finally treated with pure acetone for 20 minutes. The embedding agent treated the sample overnight, embedded the infiltrated sample, and then heated it at 70 °C overnight to obtain the embedded sample. The samples were collected using the LEICA EM UC7 ultramicrotome to obtain 90 nm sections. After staining the sections with lead citrate and uranyl acetate solution for 10 minutes, the samples were dried and imaged by TEM. Herein, bacterial treated with PBS are healthy and uniform without membrane damages, while in other groups, various phenomenons are observed, *e.g.*, Nb_2C-PVP nanosheets entered the cells (purple arrows); altered membrane damage and cell bursting (red arrows), cytoplasmic vacuolization (yellow arrows), cell membrane damage with morphological changes (blue arrows) and cell wall peeling down (dashed circles) occurred.

13. Animal model establishment.

To construct the 4T1 or CT26-bearing mice model, 1×10^6 of 4T1 cells or CT26 cells were subcutaneously injected into the mammary region or abdomen of BALB/c mice (Shanghai SLAC Laboratory Animal Co., Ltd). Meanwhile, the tumor size was measured with a Vernier caliper and the corresponding tumor volume was calculated according to the formula: volume = length $\times$width^2 $\times$0.5. Animal experiment would not be carried out until the tumor volume reached 80 mm^3. In addition, the biosafety evaluation of Nb_2C/Au/anti-TNFα-PVP was performed on female Kunming mice (4 weeks, Shanghai SLAC Laboratory Animal Co., Ltd). More importantly, mice were fed in the Laboratory

Animal Center of Shanghai Tenth Peoples' Hospital and all *in vivo* experiments were performed according to protocols approved by the Laboratory Animal Center of Shanghai Tenth Peoples' Hospital (approval number: SHDSYY-2020-3429) and were in accordance with the policies of National Ministry of Health.

14. *In vivo* toxicity assay

In vivo experiment procedures conformed to the guidelines for the Animal Care Ethics Commission of Shanghai Tenth People's Hospital, Tongji University School of Medicine. Forty-eight healthy female Kunming mice (15~20 g) were randomly divided into 4 groups (n=6) and then intravenously administered with PBS and Nb_2C/Au/anti-TNFα-PVP with varied dose concentrations ([Nb]=5 mg kg^{-1}, 10 mg kg^{-1} and 20 mg kg^{-1}). After feeding for varied days (7 days and 28 days), *in vivo* toxicity was evaluated. The body weight of mice was recorded per two days during the next 28 days. After one month, blood sample in each mouse was collected by extracting the eyeball for further blood biochemistry and blood routine analysis, including white blood cells (WBC), red blood cells (RBC), hemoglobin (HGB), mean corpuscular hemoglobin (MCH), mean corpuscular volume (MCV), platelets (PLT) and alanine transaminase (ALT), aspartate transaminase (AST), albumin (ALB), blood urea nitrogen (BUN), creatinine (CREA), alkaline phosphatase (ALP). The major organs (heart, liver, spleen, lung and kidney) were obtained, fixed in 10% paraformaldehyde, and stained with hematoxylin and eosin (H&E) for histological analysis.

15. Evaluation of anti-tumor efficacy *in vivo*

4T1 tumor-bearing mice were randomly divided into five groups (n=6), *e.g.*, Group A: control, Group B: Nb_2C/Au/anti-TNFα-PVP ([Nb] = 10 mg kg^{-1}), Group C: Nb_2CAu/anti-TNFα-PVP+1064 nm laser irradiation([Nb] = 10 mg kg^{-1}, Power density:1.0 W cm^{-2}), Group D:

Nb_2C/Au/anti-TNFα-PVP+ 1064 nm laser irradiation ([Nb] = 10 mg kg^{-1}, Power density:1.0 W cm^{-2}) (Change cage), Group E: Nb_2C/Au-PVP+1064 nm laser irradiation([Nb] = 10 mg kg^{-1}, Power density:1.0 W cm^{-2}). All *in vivo* experiments were performed according to protocols approved by the Laboratory Animal Center of Shanghai Tenth Peoples' Hospital and were in accordance with the policies of National Ministry of Health. The tumor weight and size were measured every 2 days during the following two months. The tumor volume was measured according to the following formula: (tumor length) × (tumor width)^2 × 0.5. The tumor weight was monitored within 14-day period after synergistic therapy. After that, the tumor and major organs (heart, liver, spleen, lung and kidney) were sliced and stained with H&E, TUNEL and Ki-67 for histological analysis, and also stained with anti-CD4 and anti-CD8 antibodies for immunofluorescence observation.

As for repressing CT26 tumor, identical grouping and operation procedures were carried out (n=5).

Immunofluorescence imaging-based detections of tumor microorganisms

Abundance detection of intratumoral flora was implemented based on immunofluorescence imaging. In detail, tumor tissues were collected and prepared as frozen sections in groups A-E with LPS, LTA staining for histological analysis after the corresponding treatment described above.

Detection of inflammatory cytokines by enzyme-linked immune sorbent assay (ELISA)

Blood was collected from the mice eyeball and made into serum samples after aforementioned corresponding treatment in groups A, C and E. Subsequently, the as-prepared serum samples were tested for qualifying cytokines including IL-6, IL-10, IL-12, MCP-1, IFN-r and TNFα according to the instructions of MS INFLAMMATION kit (552364BD Systems).

***In vivo* pharmacokinetics and bio-distribution of Nb2C/Au/anti-TNFα-PVP in 4T tumor and**

normal tissues

As for bio-distribution, 4T1 tumor-bearing mice were randomly divided into six groups (n=6), *e.g.*, 1 h, 3 h, 6 h, 12 h, 24 h and 48 h. Nb_2C/Au/anti-TNFα-PVP ([Nb]: 10 mg kg^{-1}) was intravenously injected into mice at = 0 h, and after 1 h, 3 h, 6 h, 12 h, 24 h and 48 h post-intravenous injection, respectively, mice were killed and tumor and normal organs including heart (H), liver (Li), spleen (Sp), lung (Lu), kidney (Ki), intestine (I) were collected for ICP-AES tests.

As for pharmacokinetics: healthy BALB/c rats (n=6) were intravenously injected with Nb_2C/Au/anti-TNFα-PVP at dosage of 4 mg/kg. 15 μL of blood was taken out at various time (0 min, 5 min, 15 min, 30 min, 1 h, 2 h, 6 h, 12 h, and 24 h) after pre-set time points post-injection and then dispersed in lysis buffer before the [Nb] concentration of each sample was measured by ICP-AES. The *in vivo* blood terminal half-life of Nb_2C/Au/anti-TNFα-PVP was calculated by a single-component pharmacokinetic model.

***In vivo* animal fluorescence imaging**

4T1 or CT26 tumor-bearing mice were intravenously injected with Nb_2C/Au/anti-TNFα-PVP labeled with Cy 5.5 (4 mg mL^{-1}, 100 μL) through the tail vein. After different time points, *in vivo* fluorescence imaging were carried out and photos and signal intensity values were recorded. As for ex vivo fluorescence imaging, after 24 post-injection of Nb_2C/Au/anti-TNFα-PVP labeled with Cy 5.5, the major organs and tumor tissues were collected for imaging.

16. Flora analysis

Tumor DNA extraction and 16S rDNA sequencing analysis

Specimen detection was completed by Shanghai Personal Biotechnology Co., Ltd. (Shanghai, China). Database was created for qualifying DNA specimens. PCR amplification was processed according to 338F_806R region. Through the quantitative detection, we built up Miseq library and performed the sequencing. PE (paired-end) acronym readings from Miseq sequencing were jointed together according to the overlapping relationship. Operational Taxonomic Unit (OTU) cluster analysis and taxonomic analysis were processed in all sequencing. We calculated the community structure of specimens mainly in phylum and genus levels.

Bioinformatics analysis

Bioinformatics analysis for PE reading from Miseq sequencing was jointed together according to the overlapping relationship. According to the degree of similarity difference, OTU cluster analysis for all sequencing was processed. We adopted a RDP classifier Bayesian algorithm to conduct taxonomic analysis and calculated each sample's community composition in each classification level. Based on OTU and its cluster analysis results as well as the taxonomic information, in-depth analysis of community structure in each classification level (such as PLS-DA), partial least squares discriminant analysis, cluster composition analysis and wilcoxon rank sum test were conducted.

17. Untargeted metabolomics analysis

Sample extraction method

The tumor tissues were rapidly frozen in liquid nitrogen immediately after dissection. When test was enforced, the tissues were then cut on dry ice (~10 mg) and placed into Eppendorf tubes (2 mL). Tissue samples were homogenized with 200 μL of H_2O and five ceramic beads using a homogenizer,

and then 800 μL of methanol/acetonitrile (1:1, v/v) was added into the homogenized solution for metabolite extraction. After the mixture was centrifuged for 15 min (14,000 g, 4 °C), the supernatant was dried in a vacuum centrifuge. For LC-MS analysis, the harvested sample was re-dissolved in 100 μL of acetonitrile/water (1:1, v/v) solvent. To monitor the stability and repeatability of instrument analysis, quality control (QC) samples were prepared by pooling 10 μL of each sample and analyzed together with the other samples. The QC samples were inserted regularly and analyzed in every 5 samples.

LC-ESI-MS/MS analysis

Analysis was performed using the ultrahigh pressure liquid chromatography (UHPLC) (1290 Infinity LC, Agilent Technologies) coupling with a quadrupole time-of-flight (AB Sciex TripleTOF 6600) in Shanghai Applied Protein Technology Co., Ltd. For hydrop interaction liquid chromatography (HILIC) separation, samples were analyzed using a 2.1 mm × 100 mm ACQUIY UPLC BEH 1.7 μm column (waters, Ireland). In both electrospray ionization (ESI) positive and negative modes, the mobile phase contained A= ammonium acetate (25 mM) and ammonium hydroxide (25 mM) in water and B= acetonitrile. The gradients follow the procedures: kept in 85% B for 1 min, linearly reduced to 65% in 11 min, reduced to 40% in 0.1 min and kept for 4 min, increased to 85% in 0.1 min, and ultimately maintained re-equilibration within 5 min. To realize reversed phase liquid chromatography (RPLC) separation, a 2.1 mm × 100 mm ACQUIY UPLC HSS T3 1.8 μm column (waters, Ireland) was used. In ESI positive mode, the mobile phase contained A= water with 0.1% formic acid and B= acetonitrile with 0.1% formic acid; while in ESI negative mode, the mobile phase contained A=0.5 mM ammonium fluoride in water and B= acetonitrile. The gradients follow the procedures: 1% B for 1.5 min, linearly increased to 99% in 11.5 min and kept for 3.5 min.

Afterwards, it was reduced to 1% in 0.1 min, and subsequently, a 3.4 min of re-equilibration period was employed. The gradients were carried out at a flow rate of 0.3 mL/min, and the column temperatures were kept constant at 25 °C, wherein a 2 μL aliquot of each sample was injected. The ESI source conditions were set as follows: Ion Source Gas1 (Gas1) as 60, Ion Source Gas2 (Gas2) as 60, curtain gas (CUR) as 30, source temperature: 600 °C, IonSpray Voltage Floating (ISVF) ± 5500 V. In mass spectrometer (MS) acquisition alone, the instrument was set to acquire across the m/z range 60-1000 Da, and the accumulation time for TOF MS scan was set at 0.20 s/spectra. In auto MS/MS acquisition, the instrument was set to acquire over the m/z range 25-1000 Da, and the accumulation time for product ion scan was set at 0.05 s/spectra. The product ion scan was acquired using information dependent acquisition (IDA) with high sensitivity mode. The parameters were set as follows: the fixed collision energy (CE) at 35 V with ± 15 eV; declustering potential (DP) at 60 V (+) and −60 V (−); the excluding isotopes within 4 Da; the monitoring candidate ions per cycle: 10.

Data processing

The raw MS data (wiff. scan files) were converted to MzXML files using ProteoWizard MS Convert before importing into freely available XCMS software. For peak picking, the following parameters were used: centWave m/z = 25 ppm, peakwidth = c (10, 60), prefilter = c (10, 100). For peak grouping, bw = 5, mzwid = 0.025, minfrac = 0.5 were used. CAMERA (Collection of Algorithms of MEtabolite pRofile Annotation) was used for annotating isotopes and adducts. In the extracted ion features, only the variables that possessed more than 50% of the nonzero measurement values in at least one group were kept. Compound identification of metabolites was performed by comparing the accuracy of m/z value (< 25 ppm), and MS/MS spectra with an in-house database were established *via* referring to the available authentic standards.

Each bubble in the bubble diagram represents a metabolic pathway, where the top 20 pathways with high significances were exhibited according to the P value. The horizontal axis and size of the bubbles indicate the influencing factor level of such a pathway in the topological analysis, where the larger bubble size means the larger influencing factor. The impact-value threshold that was calculated from the pathway topology analysis was set to 0.10. The vertical coordinate and color of bubbles represent the P-value level of the enrichment analysis (using the negative common logarithm, *i.e.*, -log10 P-value), and the darker color means the smaller P-value, suggesting larger significance of the enrichment degree. According to the metabolic pathway analysis, the significant differences between groups A and C are alanine, aspartic acid and glutamate metabolisms, pyrimidine metabolism, unsaturated fatty acids biosynthesis and purine metabolism, and between groups A and D, nucleotide metabolism difference is dominant. Notably, there are the significant pathway differences between groups D and C in glutamatergic synapse, GABAergic synapse, D-arginine and D-ornithine metabolism, D-glutamine and D-glutamate metabolism, proximal tubule bicarbonate reclamation, nitrogen metabolism, arginine biosynthesis, pyrimidine metabolism, purine metabolism and ABC transporter proteins.

18. Statistical analysis.

The data were derived from at least three independent experiments and all obtained data were expressed as mean value ± standard deviation (SD) (n=3-6). Statistical analysis was performed by using Origin 8.0 software and SPSS software, and a two-tailed t-test method was used. After normalization of the total peak intensity, the untargeted metabolic analysis was performed on the processed data where the R package (ropls) was used for the multivariate data analysis including Pareto-scale principal component analysis (PCA) and orthogonal partial least squares discriminant

analysis (OPLS-DA). Seven-fold cross-validation and response permutation tests were used to evaluate the robustness of the model. The values of variable importance for the projection (VIP) were calculated for each variable in the OPLS-DA model in the projection to indicate its contribution to the classification. Metabolites with VIP values >1 were further measured by applying Student's t-test at the univariate level to indicate the significance of each metabolite. Single, double and triple asterisks represent $P < 0.05$, 0.01 and 0.001, respectively, and *$P < 0.05$ was considered statistically significant and **$p < 0.01$ was extremely significant.

Part B: *Supplementary Tables*

Table S1 Various pathway metabolites identified by UHPLC−QTOF-MS and their expression levels by comparing C and A groups in positive or negative ionization mode.

adduct	metabolite	m/z	rt (s)	p-value	FC	VIP
(M+Na)+	Thioetheramide-PC	758.56982	115.136	0.02135165	0.737748338	13.32758784
M+	2-Methylbutyroylcarnitine	246.17047	236.237	0.028050774	3.09707782	7.997830239
(M+H)+	N6,N6,N6-Trimethyl-L-lysine	189.15811	538.797	0.031754465	2.181157737	2.003072426
(M+H)+	Val-Thr	219.13498	51.922	0.032761058	2.166148322	1.213110174
(M+H)+	1-Stearoyl-2-hydroxy-sn-glycero-3-phosphocholine	524.37092	186.235	0.035022355	1.31264529	13.06740155
(M+H-H_2O)+	D-Ornithine	115.08536	203.874	0.035704028	2.336692116	1.100831526
(M-2H+3Na)+	3'-O-methylcytidine	324.06052	446.743	0.040077448	1.899868813	1.139318329
(M+H)+	L-Palmitoylcarnitine	400.34194	169.497	0.04485329	2.193549089	13.21082351
(M+Na)+	Eicosapentaenoic acid	325.21451	70.675	0.046351588	2.147182883	1.305358481
(M+Na)+	16-Hydroxypalmitic acid	295.2252	70.822	0.046621901	2.311587503	1.396165066
(M-H+2Na)+	L-Glutamine	191.03898	375.387	0.048774938	1.901398216	1.017302718
(M-H)-	Thymine	125.03584	76.336	0.012754741	1.566968811	3.633478524
(2M-H)-	Arachidonic Acid (peroxide free)	607.4705	40.266	0.014464053	1.53118302	2.06552396
(2M-H)-	3,3',4,5'-Tetrahydroxy-trans-stilbene	487.13047	162.886	0.015843017	0.281430093	3.72994009
(M-H)-	Heptadecanoic acid	269.24721	79.652	0.016252653	1.778742868	1.282364861
(M-H)-	9R,10S-EpOME	295.22771	57.317	0.017122882	1.520761612	7.459907676
(M-H)-	Hypoxanthine	135.0315	198.258	0.020176644	0.563925247	7.877907958
(M-H)-	Taurochenodeoxycholate	498.28845	155.3465	0.020829716	1.96680085	1.817234827
(M-H)-	3-Hydroxycapric acid	187.13382	104.481	0.021879681	2.280374387	2.155248044
(M-H)-	N-Acetyl-L-aspartic acid	174.04122	400.953	0.023131079	1.849585345	3.295536739
(M-H)-	D-Mannose 1-phosphate	259.02226	501.619	0.024086893	0.834829137	1.737176101

Note, m/z means mass-to-charge ratio; FC means fold change; rt means tetention time; VIP means variable importance in projection

Table S2 Various pathway metabolites identified by UHPLC−QTOF-MS and their expression levels by comparing D and A groups in positive or negative ionization mode.

adduct	metabolite	m/z	rt (s)	p-value	FC	VIP
(M+H)+	DL-Arginine	175.11796	580.826	0.050308011	0.563156457	<1
(M+H)+	Urocanic acid	139.04939	290.907	0.052062513	3.365917251	<1
(M+H)+	1-Myristoyl-sn-glycero-3-phosphocholine	468.30751	193.803	0.065089557	0.680987088	<1
(M+H)+	Adenine	136.06062	167.59	0.06567989	1.82046409	<1
(M+H)+	Cytidine	244.092	238.73	0.073666066	0.658033745	<1
(M-H+2Na)+	3-Methylhistamine	115.08536	203.874	0.035704028	2.647947127	<1
(M+H)+	3-Methylhistidine	324.06052	446.743	0.040077448	0.54041344	<1
(M-H)-	15-Deoxy-delta-12,14-PGJ2	315.1953	85.87	0.01489486	0.590721163	2.348248888
(M-H)-	Hypoxanthine	135.0315	198.258	0.024450449	0.538120974	7.702501158
(M-H)-	Lumichrome	241.07242	62.12	0.026903009	0.638034969	1.892205266
(M-H)-	Thymidine	241.08285	101.715	0.035452964	0.67597527	2.648358983
(M-H)-	Urocanic acid	137.03575	292.29	0.046405501	3.487390915	1.093900891

Table S3 Various pathway metabolites identified by UHPLC-QTOF-MS and their expression levels by comparing C and D groups in positive or negative ionization mode.

Adduct	Metabolite	m/z	rt (s)	p-value	FC	VIP
(M+H-H_2O)+	D-Ornithine	115.08536	203.874	0.016887552	0.342149488	1.264681693
(M+H)+	Cytosine	112.04968	205.779	0.025656404	0.550074589	2.413360012
(M-H+2Na)+	L-Glutamine	191.03898	375.387	0.028342057	0.42243673	1.214650402
(M+H)+	1-methylguanosine	298.11337	196.319	0.028844167	0.567438876	1.140052468
(M+H)+	Cytidine	244.092	238.73	0.031557339	0.407957725	2.236365535
(M+H)+	N6,N6,N6-Trimethyl-L-lysine	189.15811	538.797	0.036600678	0.468311572	1.883469206
M+	2-Methylbutyroylcarnitine	246.17047	236.237	0.03937116	0.363899929	9.610470993
(M+H)+	Adenine	136.06062	167.59	0.044197947	1.95900847	2.865004324
(M+H)+	Trimethylamine N-oxide	76.0753	327.437	0.046985465	0.610531981	1.615593504
(M+H)+	Adenosine3'-monophosphate	348.06911	409.716	0.049800765	0.628562844	1.852960514

Part C: *Supplementary Figures*

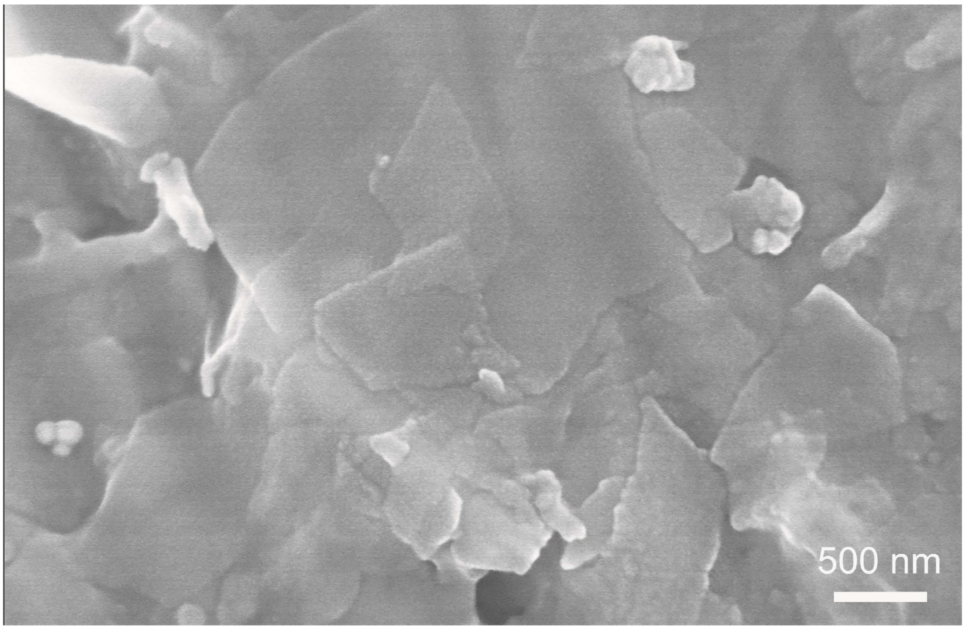

Figure S1. SEM image of multi-layer Nb_2C nanosheets obtained by HF selective etching of $NbAl_2C$.

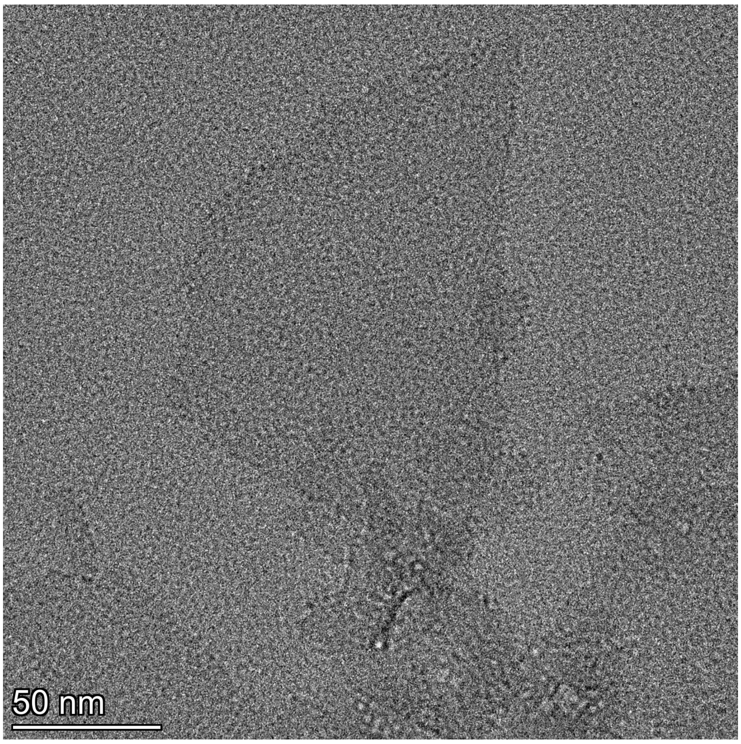

Figure S2. TEM image of Nb_2C nanosheets with single layer structure.

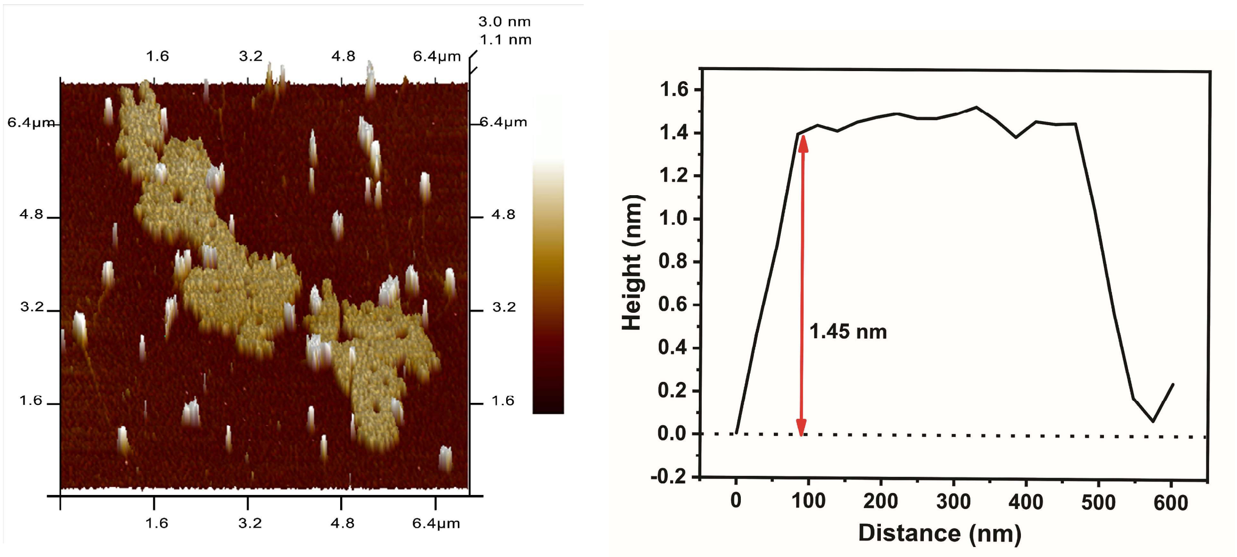

Figure S3. Atom force microscopy image of Nb_2C nanosheets for determining the thickness.

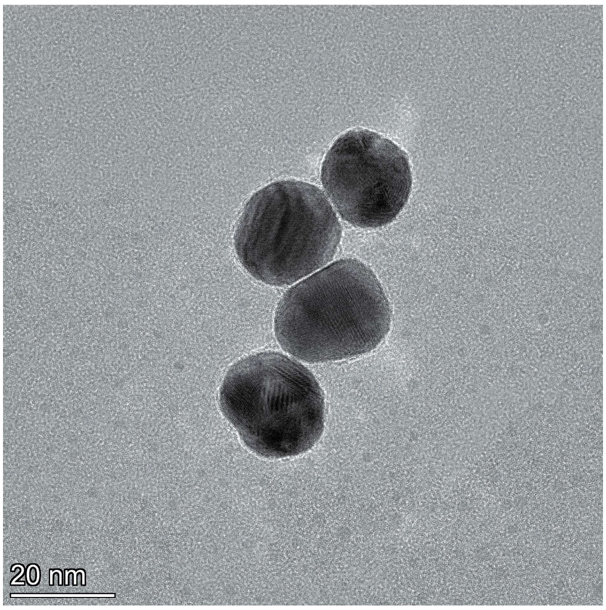

Figure S4. TEM image of Au nanoparticles.

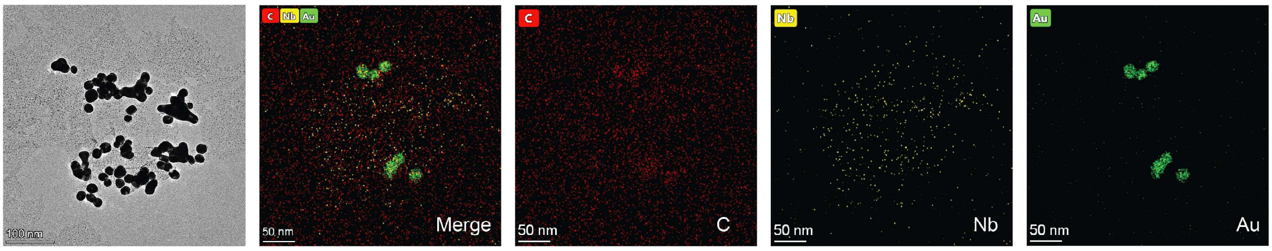

Figure S5. SEM and corresponding elemental mapping of Nb_2C/Au for observing the distributions of these atoms (Nb, Au and C).

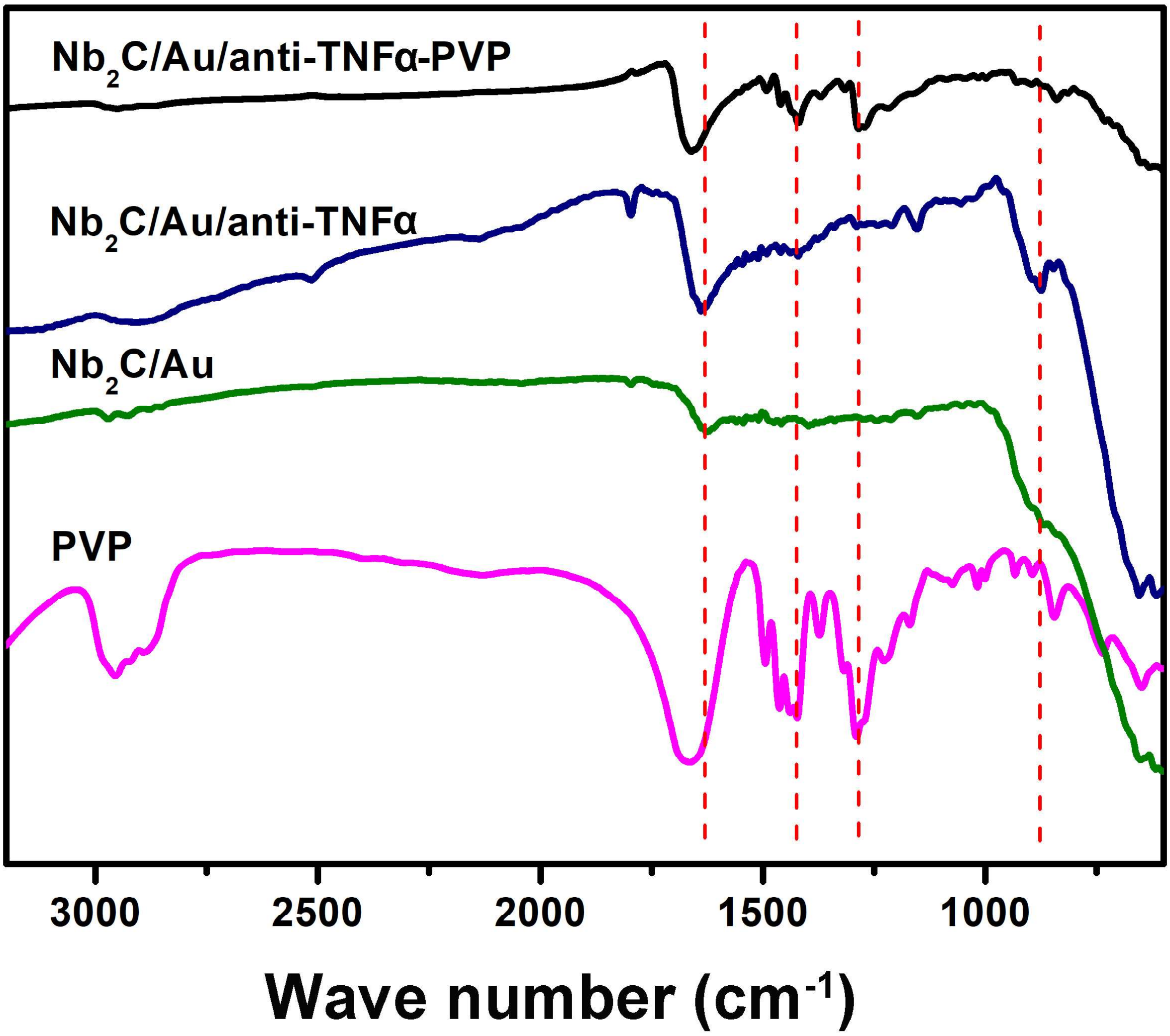

Figure S6. Fourier transform infrared spectroscopy (FTIR) spectra of PVP, Nb_2C/Au, Nb_2C/Au/anti-TNFα and Nb_2C/Au/anti-TNFα-PVP.

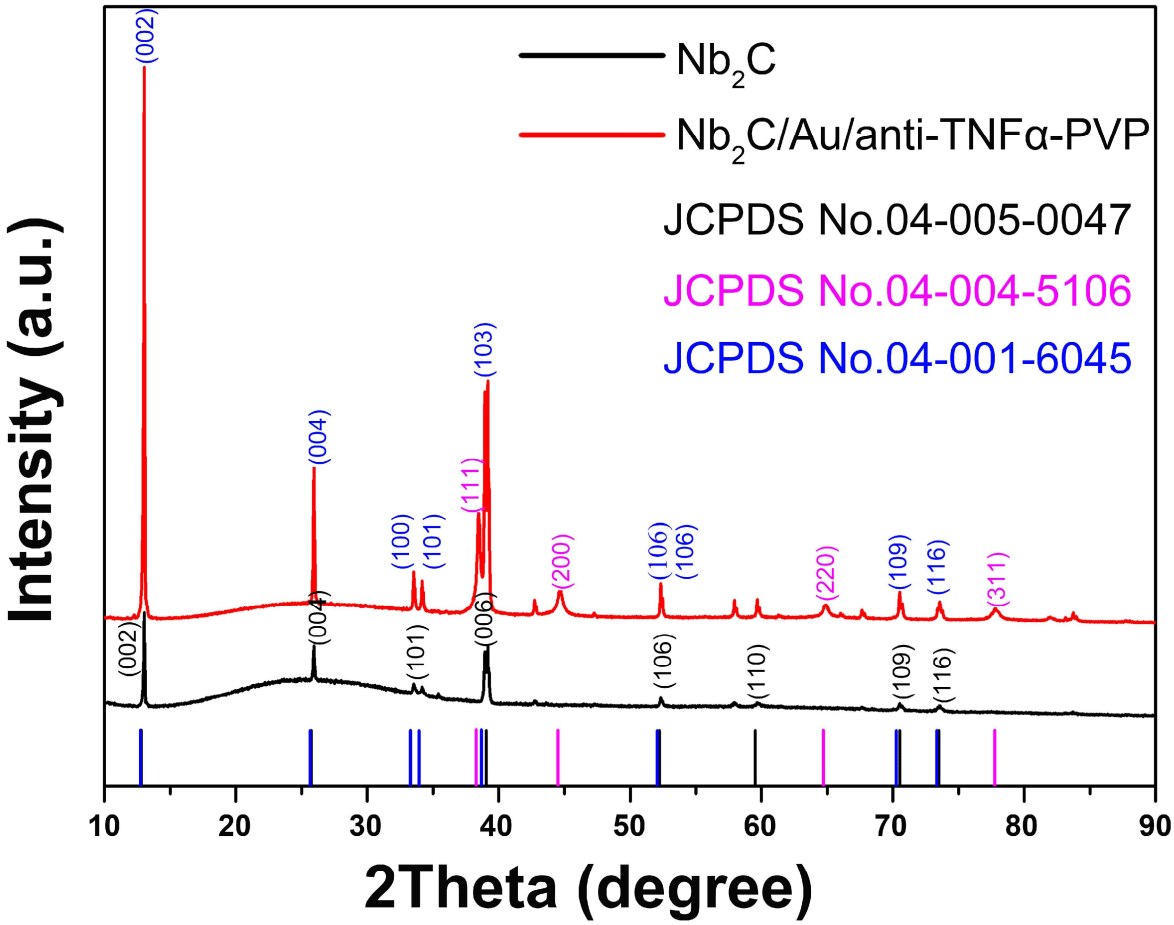

Figure S7. X-Ray diffraction (XRD) patterns of Nb_2C nanosheets and Nb_2C/Au/anti-TNFα-PVP nanoplatforms, where no evident peak shift suggests no alteration of crystalline structure after Au and anti-TNFα co-encapsulation.

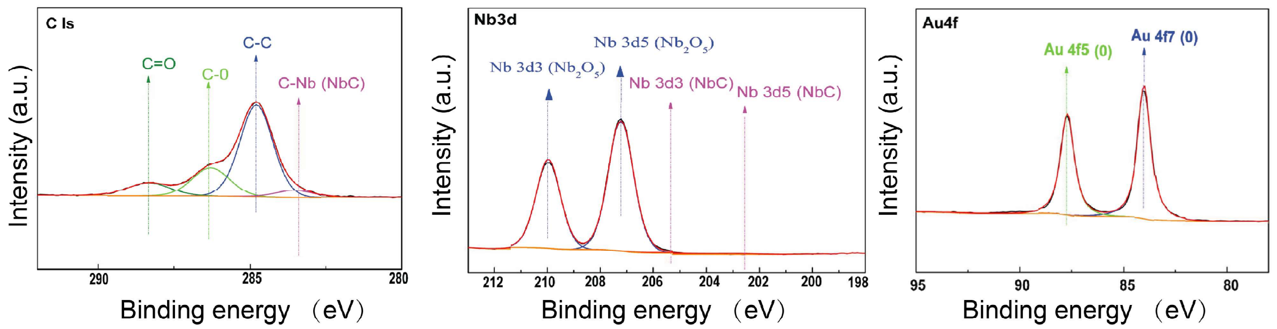

Figure S8. X-ray photoelectron spectroscopy (XPS) spectra of C1s, Nb3d and Au4f in Nb_2C/Au/anti-TNFα-PVP.

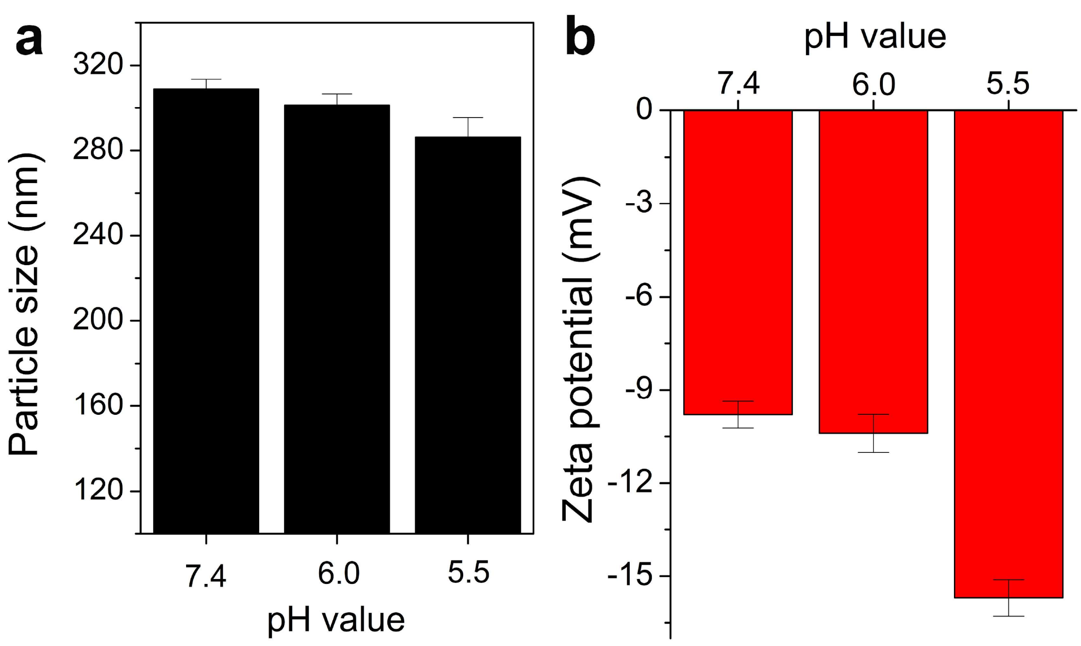

Figure S9. (a) Particle sizes and zeta potentials of Nb_2C/Au/anti-TNFα-PVP in sodium citrate-hydrochloric acid buffer solution with varied pH values (7.4, 6.0 and 5.5). Data are expressed as mean ± SD (n=3).

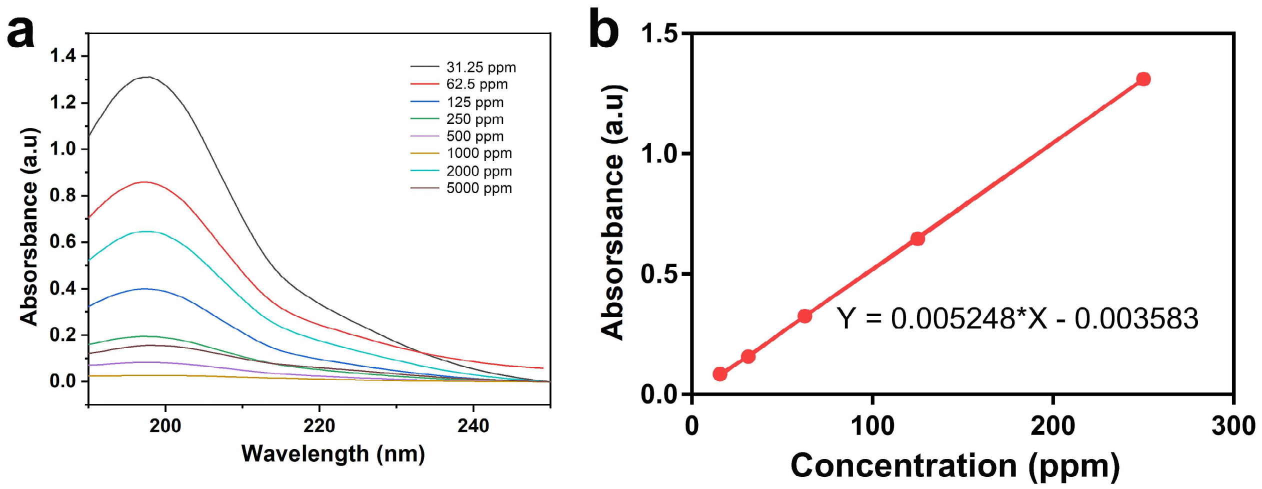

Figure S10. (a) UV-vis spectra of anti-TNFα with varied concentrations; (b) The standard curve of anti-TNFα concentration to absorbance intensity acquired at the wavelength of 200 nm.

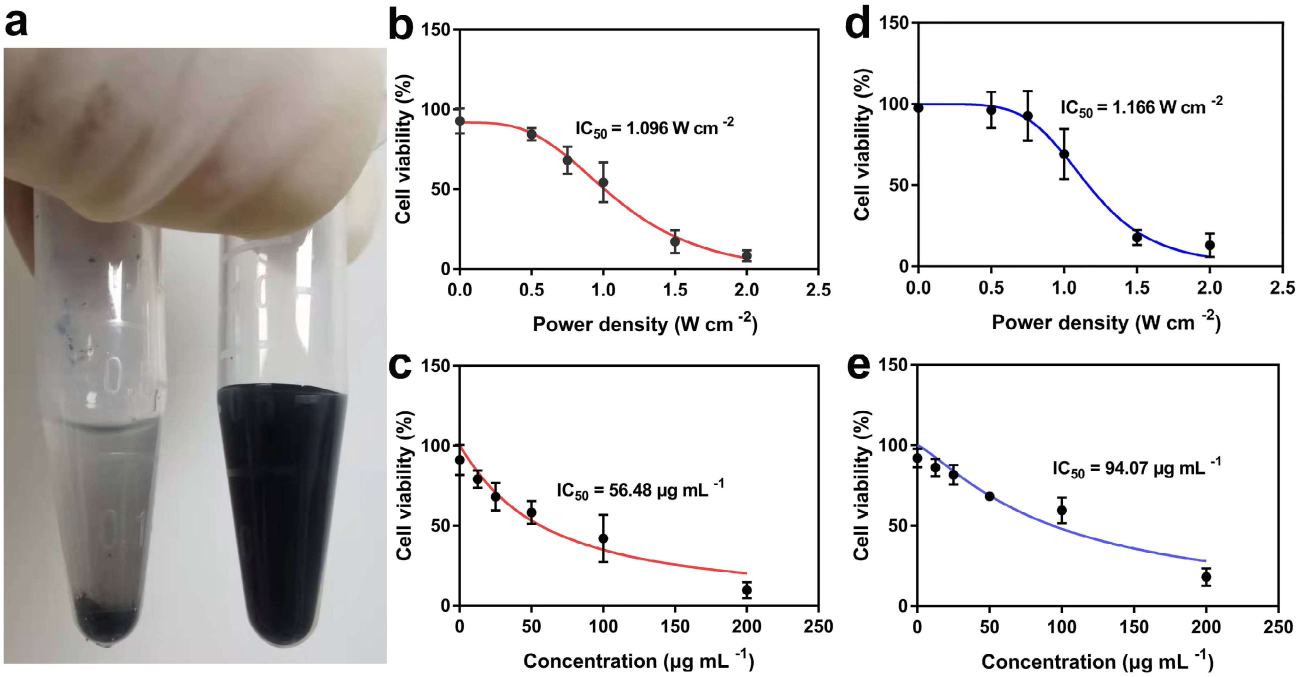

Figure S11. (a) Digital photo of Nb_2C/Au/anti-TNFα (left) and Nb_2C/Au/anti-TNFα-PVP (right) in PBS solution for 1 h. (b,d) Viabilities of 4T1 after incubation with PVP-modified Nb_2C/Au (b) and PVP-modified Nb_2C (d) in the presence of different power densities for determining IC_{50} of power density at a fixed irradiation time of 5 min and fixed Nb concentration of 100 μg mL^{-1}. (c,e) Viabilities of 4T1 after incubation with PVP-modified Nb_2C/Au (c) and PVP-modified Nb_2C (e) with varied Nb concentrations for determining IC_{50} of Nb concentration at a fixed irradiation time of 5 min and fixed power density of 0.75 W cm^{-2}. Data are expressed as mean ± SD (n=3)

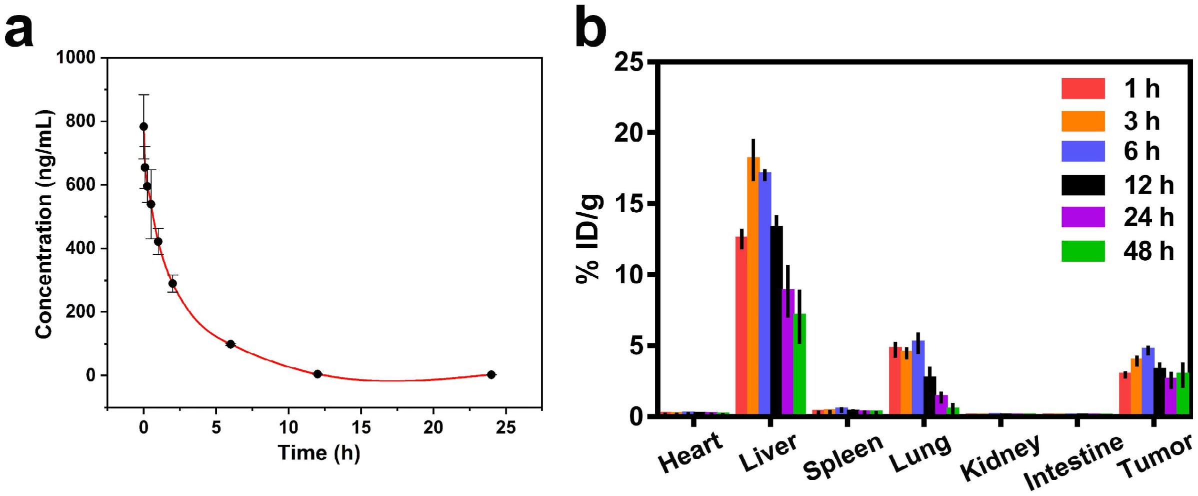

Figure S12. (a) Time-dependent concentrations of Nb in blood of 4T1-bearing mice for monitoring the half-life. (b) Time-dependent distributions of Nb_2C/Au/anti-TNFα-PVP in tumor and other normal organs after intravenously injecting Nb_2C/Au/anti-TNFα-PVP. Data are expressed as mean ± SD (n=6).

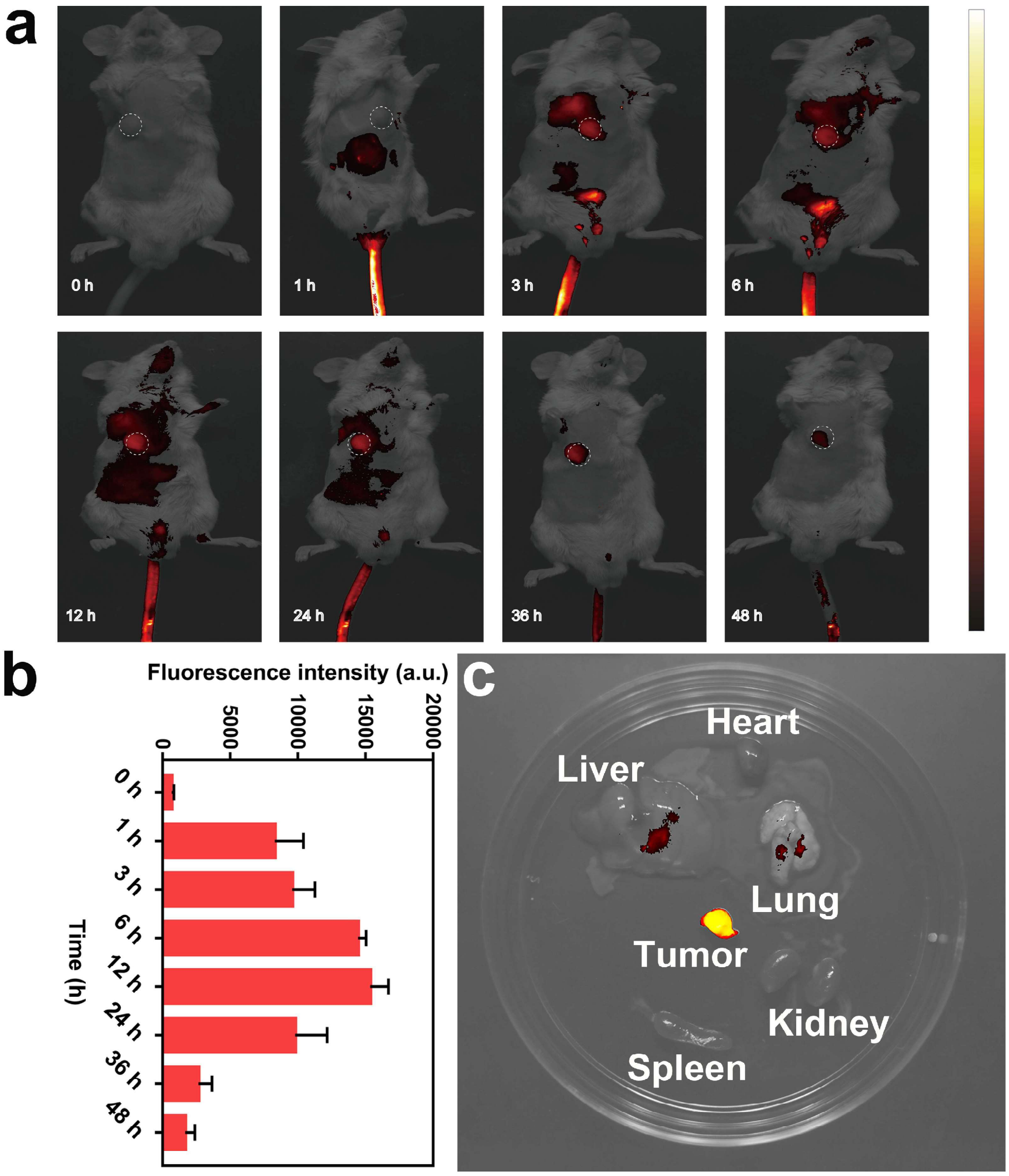

Figure S13. (a,b) Time-dependent *in vivo* fluorescence images (a) and corresponding fluorescence intensities (b) of 4T1-bearing BALB/c mice that were intravenously injected with Nb_2C/Au/anti-TNFα-PVP at dosage of 4 mg/kg, wherein dotted circles indicate the tumor; (c) *Ex vivo* fluorescence images of normal organs and tumor harvested above mice. Data are expressed as mean ± SD (n=3).

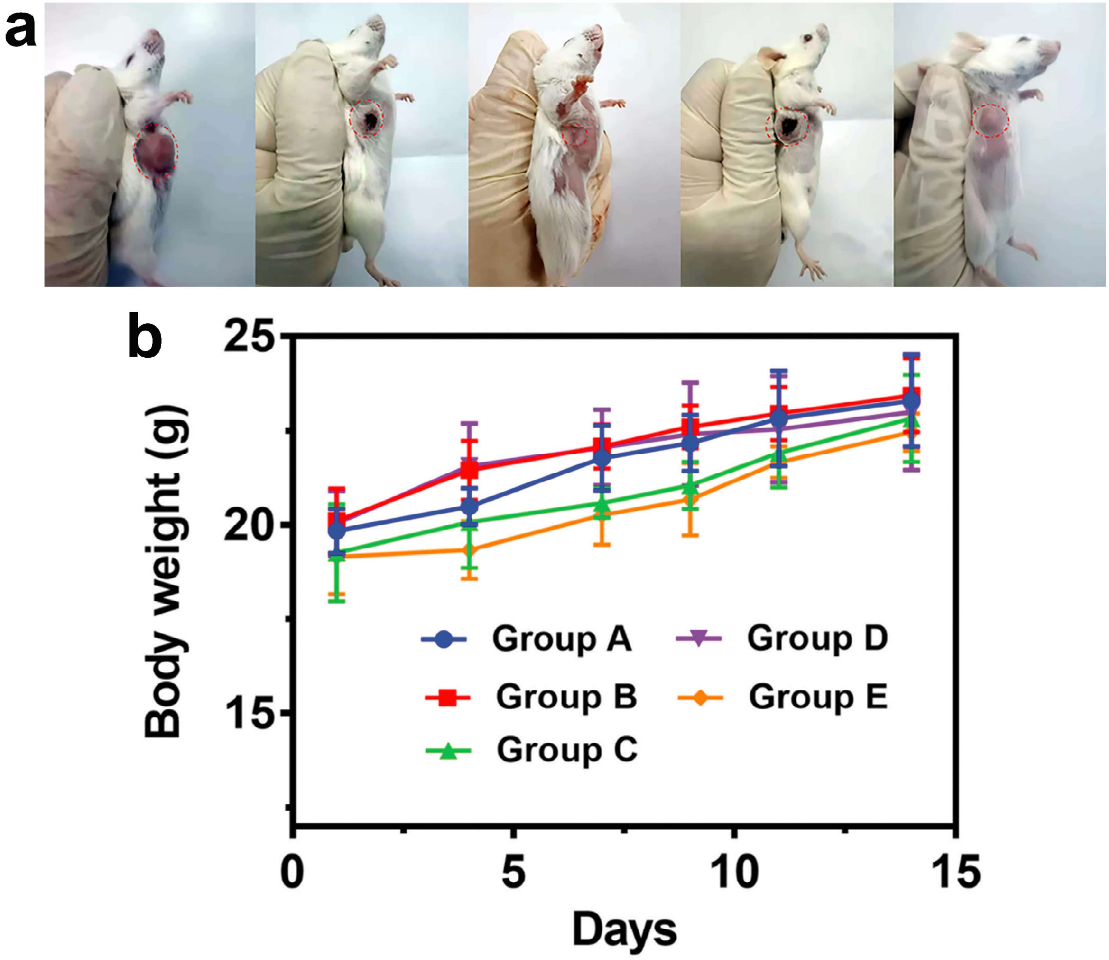

Figure S14. (a) Representative digital photos of 4T1 breast tumors at the end of monitoring period in 4T1 breast tumors-bearing babl/c mice model that experienced different treatments in different groups. (b) Time-dependent weight variation profiles of 4T1 breast tumors-bearing babl/c mice. Note, Groups A-E represent Control, laser alone, Nb_2C/Au/anti-TNFα-PVP+laser, Nb_2C/Au /anti-TNFα-PVP+laser (Control group Stool setting), Nb_2C/Au-PVP+laser, respectively; and the parameters of laser irradiations were set as: power density - 0.75 W cm^{-2}, duration - 6 min. Data are expressed mean ± SD (n=6).

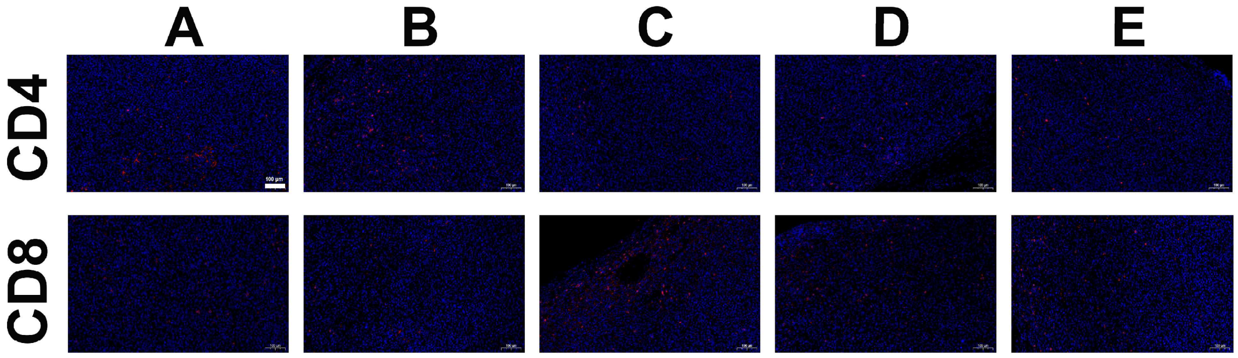

Figure S15. Immunofluorescence images of anti-CD4 and anti-CD8 antibodies-stained 4T1 tumor slices harvested from 4T1-bearing mice that experienced different treatments in groups A-E. Note, groups A-E represent, respectively, and red fluorescence indicate CD4+ and CD8+ T cells.

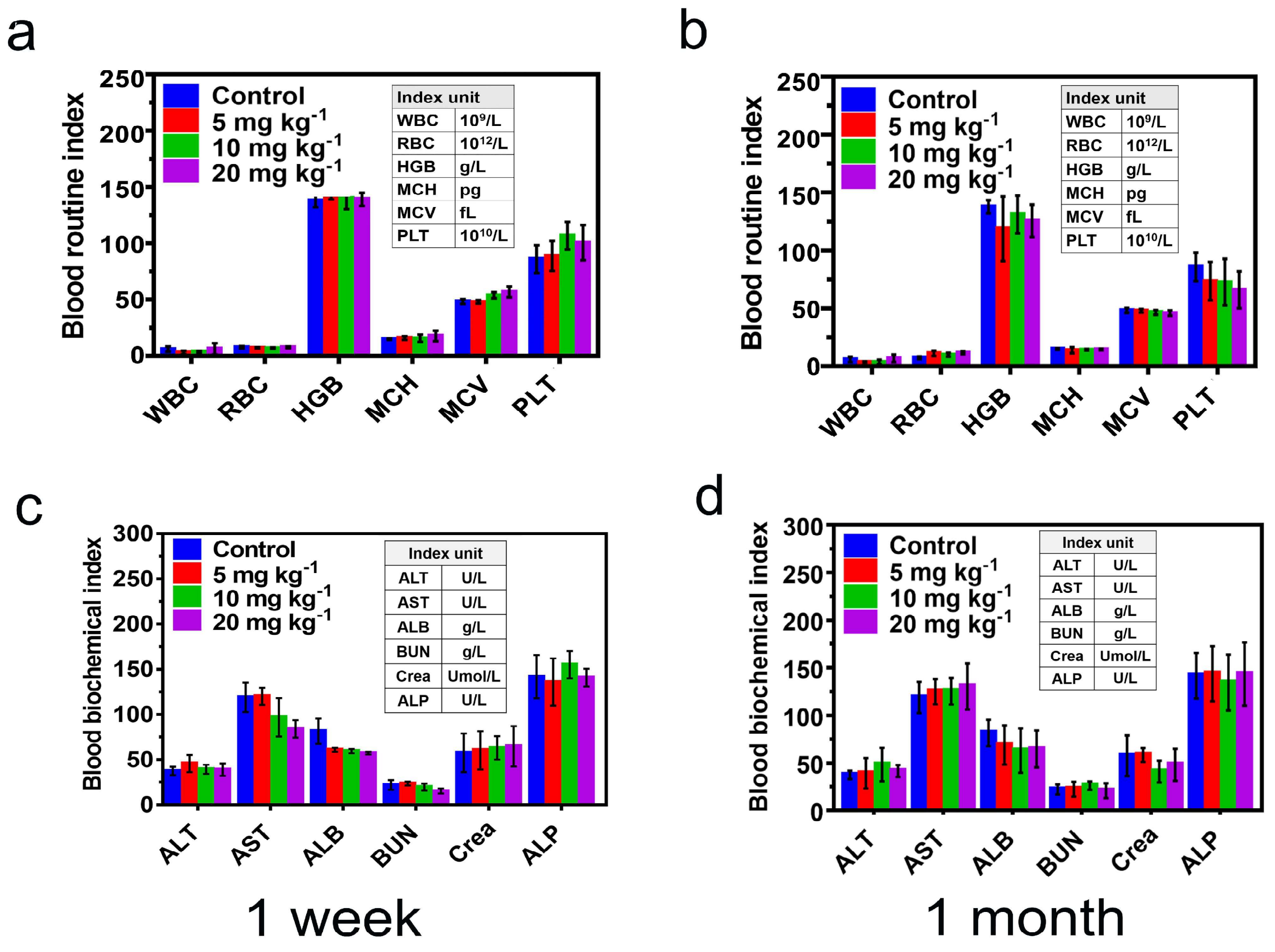

Figure S16. Hematological indexes and biochemical blood indexes of mice after 1 week (a,c) and 1 month (b,d) post-injections with Nb_2C/Au/anti-TNFα-PVP with different doses ([Nb] = 0, 5, 10, 20 mg kg^{-1}). These indexes include white blood cells (WBC), red blood cells (RBC), hemoglobin (HGB), mean corpuscular hemoglobin (MCH), mean corpuscular volume (MCV), platelets (PLT), alanine aminotransferase (ALT), aspartate transaminase (AST), albumin (ALB), blood urea nitrogen (BUN), creatinine (Crea) and alkaline phosphatase (ALP). Data are expressed mean ± standard deviation (SD) (n=6).

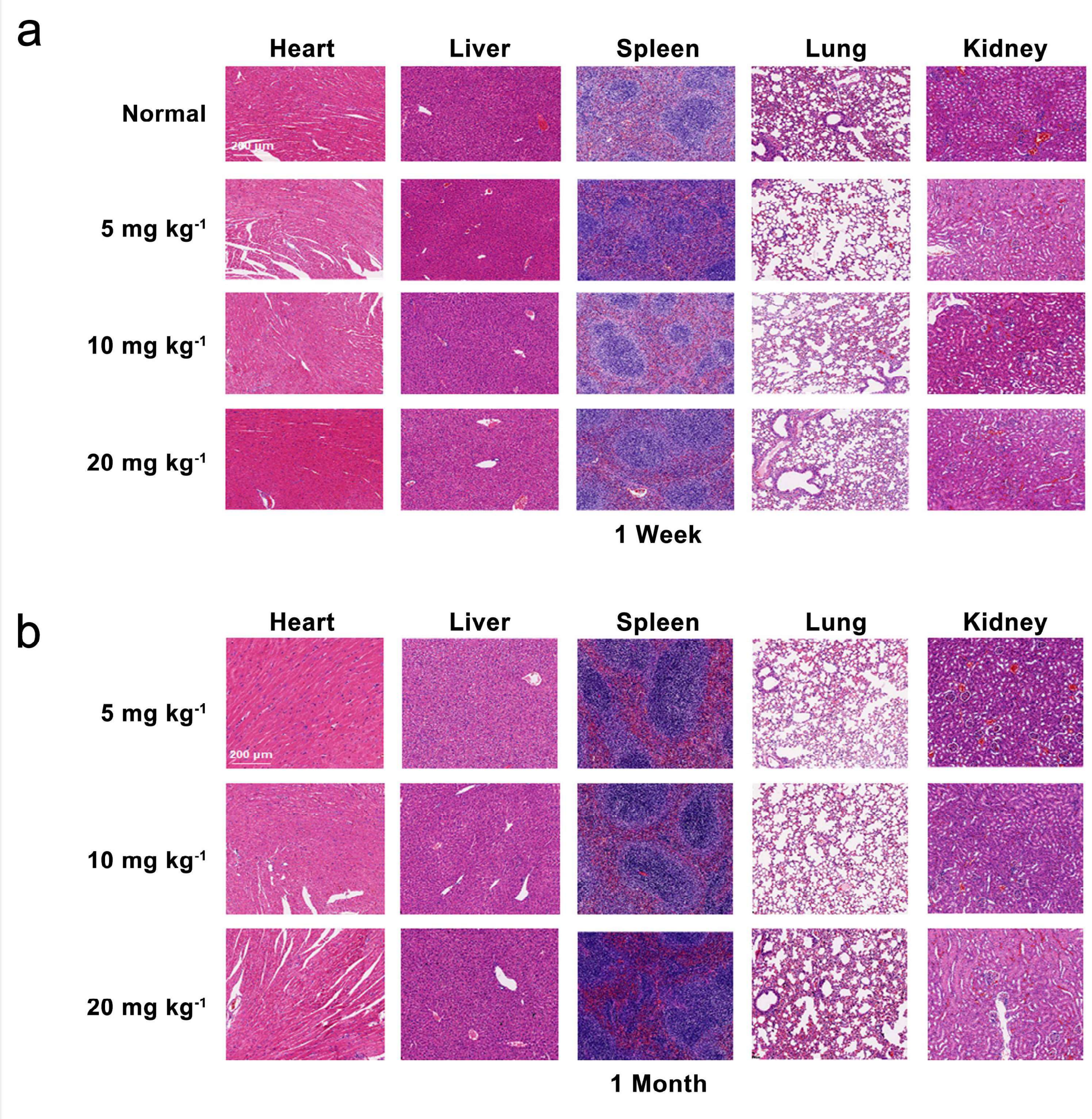

Figure S17. Optical microscopic images of major organs (heart, liver, spleen, lungs and kidneys) stained with hematoxylin and eosin (H&E) harvested from mice after 1 week and 1 month post-injections of Nb_2C/Au/anti-TNFα-PVP with different doses (Scale bar: 200 μm).

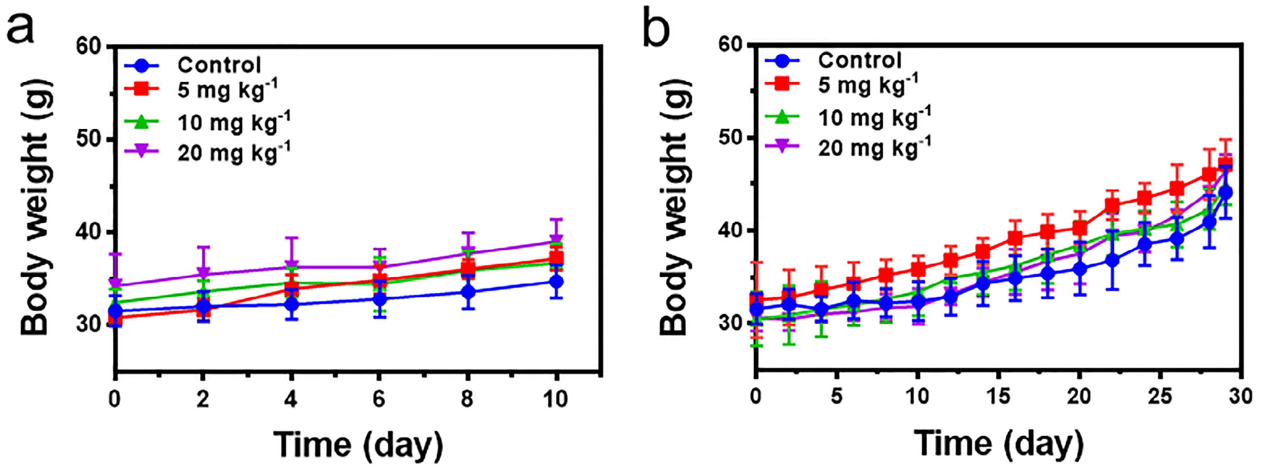

Figure S18. Time-dependent body-weight curves of nude mice after 1 week (a) and 1 month (b) post-injections of Nb_2C/Au/anti-TNFα-PVP with varied doses ([Nb] = 0, 5, 10, 20 mg kg $^{-1}$). Data are expressed mean ± SD (n=6).

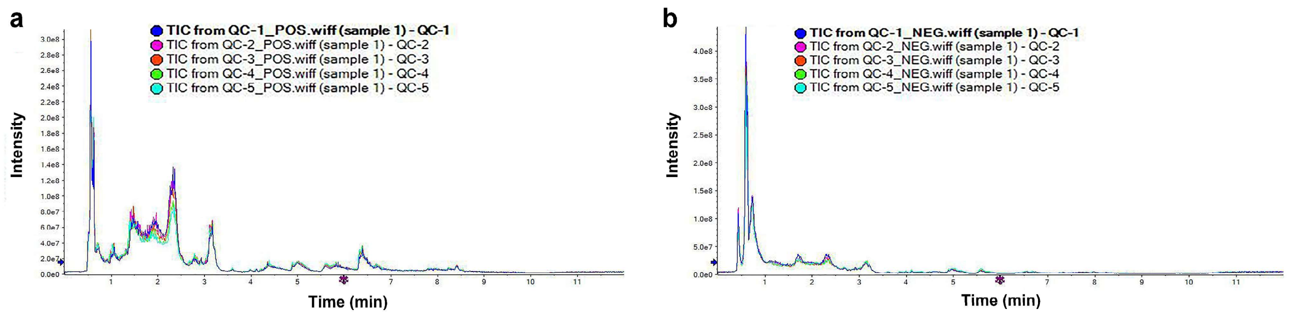

Figure S19. Total ion overlapping chromatogram spectra of quality control (QC) samples in positive (POS (a) and NEG (b). According to the total ion chromatogram (TIC) of QC samples, the response intensities and retention time of the chromatographic peaks are basically overlapped, indicating that the variation caused by the instrument error is trivial during the entire experiment. Note: The horizontal axis represents the retention time of each peak, and the vertical axis represents the intensity value of peaks.

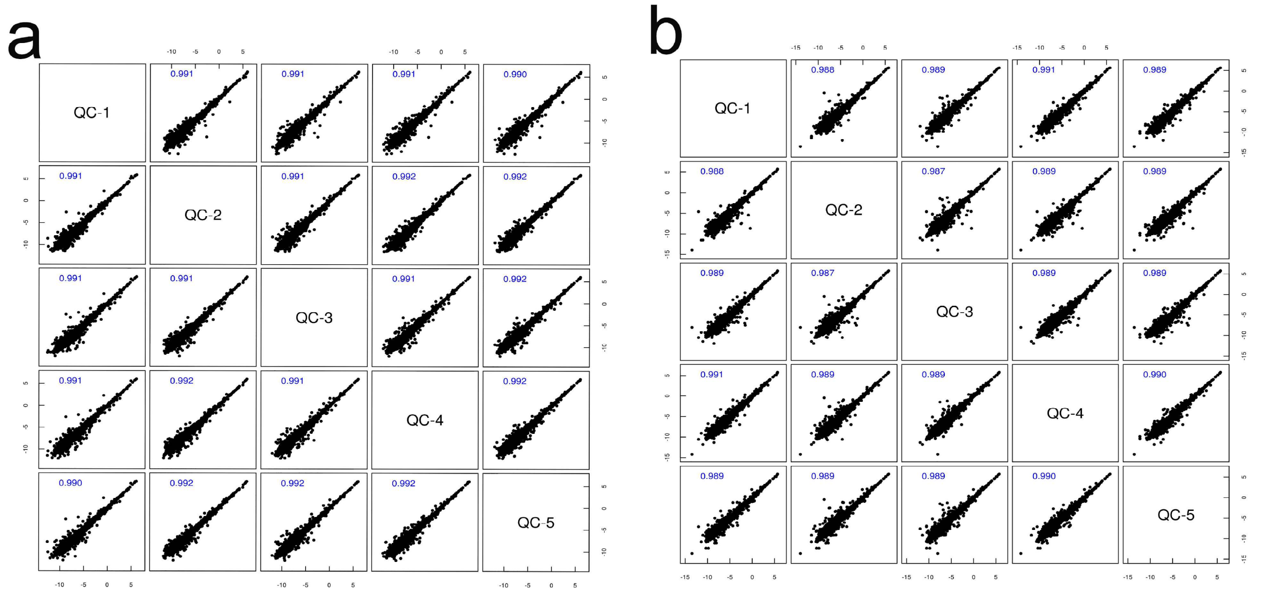

Figure S20. The correlation mappings of QC samples in both positive (a) and negative (b) ion patterns during Perform Pearson correlation analysis. Results show that the correlation coefficients of QC samples are above 0.9, indicating that the experiments feature high repeatability.

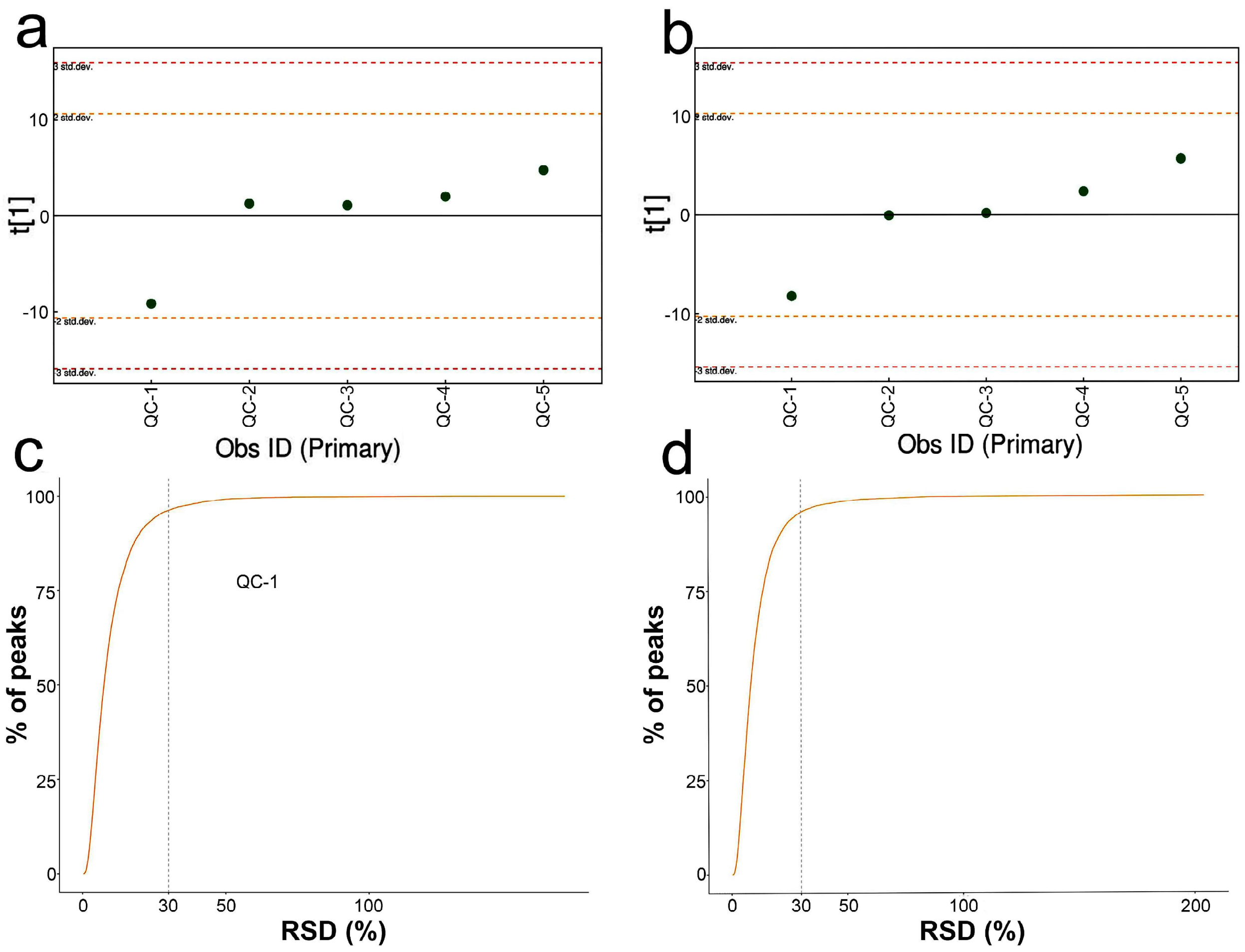

Figure S21. (a,b) Multivariate control chart (MCC) images of different QC samples in both positive (POS) ion mode (a) and negative (NEG) ion mode (b); and (c,d) relative standard deviation (RSD) of different QC samples (QC-1-QC-5) in both POS ion mode (c) and NEG ion mode (d). The horizontal (X) axis represents QC samples, and the vertical (Y) axis reflects the standard deviation. Note, the yellow and red lines define the range of standard deviations, *i.e.*, ±2 and ±3, respectively. According to MMC image (a,b), the fluctuation of QC samples within ±3 standard deviation suggest that the fluctuation of instrument is within the normal range. According to RSD image (c,d), the number of peaks with RSD ≤ 30% in the QC samples accounts for more than 80% of the total peaks, indicating that the instrument analysis system is highly stable and the data can be used for subsequent analysis.

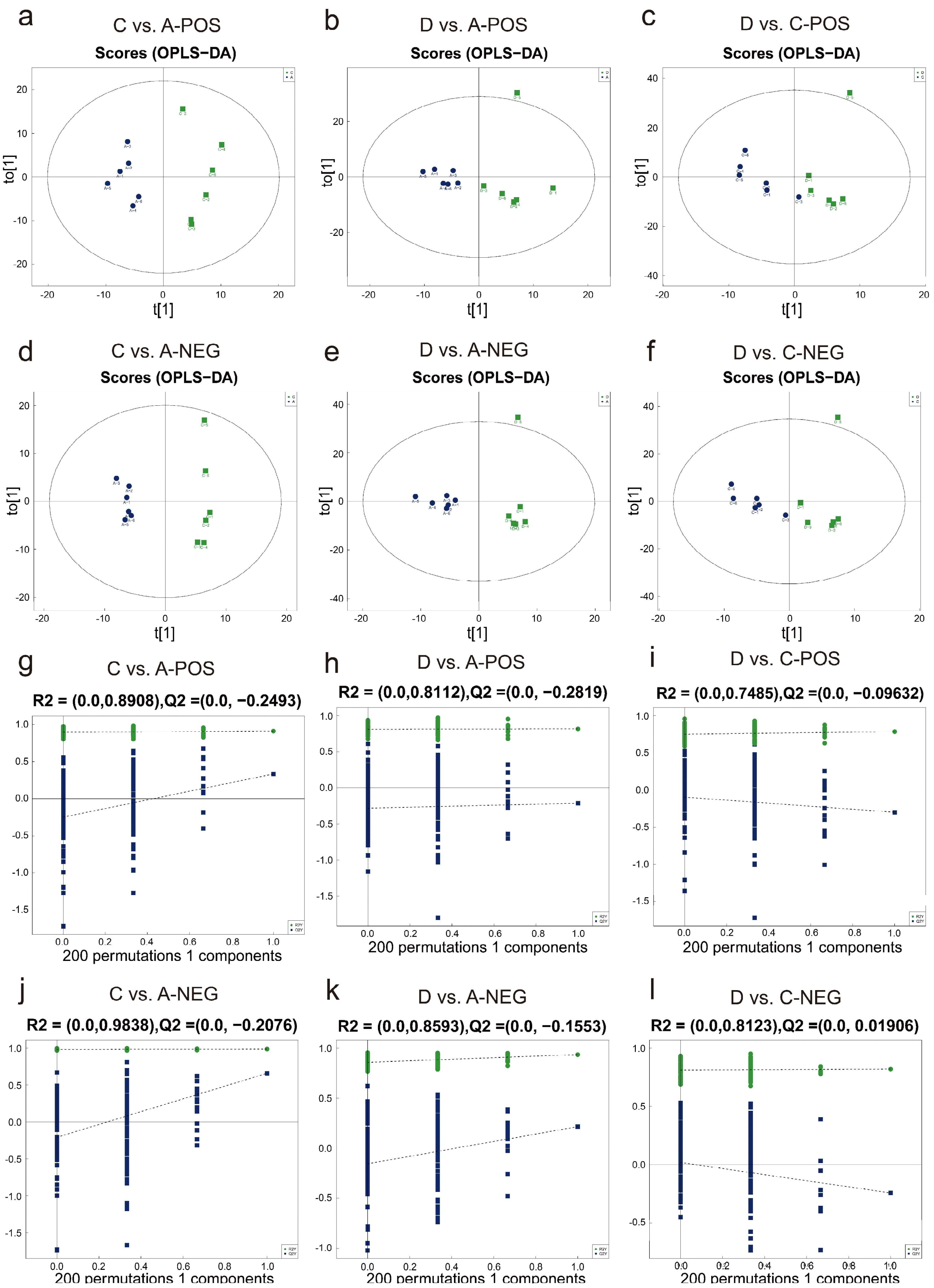
a
C vs. A-POS
Scores (OPLS−DA)
to[1]
t[1]
b
D vs. A-POS
Scores (OPLS−DA)
to[1]
t[1]
c
D vs. C-POS
Scores (OPLS−DA)
to[1]
t[1]
d
C vs. A-NEG
Scores (OPLS−DA)
to[1]
t[1]
e
D vs. A-NEG
Scores (OPLS−DA)
to[1]
t[1]
f
D vs. C-NEG
Scores (OPLS−DA)
to[1]
t[1]
g
C vs. A-POS
R2 = (0.0,0.8908),Q2 =(0.0, −0.2493)
200 permutations 1 components
h
D vs. A-POS
R2 = (0.0,0.8112),Q2 =(0.0, −0.2819)
200 permutations 1 components
i
D vs. C-POS
R2 = (0.0,0.7485),Q2 =(0.0, −0.09632)
200 permutations 1 components
j
C vs. A-NEG
R2 = (0.0,0.9838),Q2 =(0.0, −0.2076)
200 permutations 1 components
k
D vs. A-NEG
R2 = (0.0,0.8593),Q2 =(0.0, −0.1553)
200 permutations 1 components
l
D vs. C-NEG
R2 = (0.0,0.8123),Q2 =(0.0, 0.01906)
200 permutations 1 components

Figure S22. Quality control *via* establishing OPLS-DA patterns. (a-f) OPLS-DA score plots between groups A and C (a,d), A and D (b,e), and C and D (c,f) in both positive (POS) (a-c) and negative (NEG) (d-f) ion modes, wherein no sample overlapping between these groups were distinguished when the parameters Q2 were greater than 0.5, suggesting the predictive power and notable discrepancies of the patterns. (g-i) OPLS-DA permutation test chart for estimating the OPLS-DA patterns and ensuring the model validity. As the permutation retention gradually decreases, R2 and Q2 values of the random model gradually drop, indicating that the original model has no over-fitting phenomenon and the model is equipped with good robustness. The replacement test shows that the model has good repeatability and the predictability is also within an acceptable range. Note: Groups A-D represent Control, Laser alone, Nb_2C/Au/anti-TNFα-PVP+Laser, Nb_2C/Au/anti-TNFα-PVP+Laser (cage change), respectively, and in Group D, the treatment method was identical to that of Group C, but after treatment, mice were transferred to the fecal environment of tumor-bearing mice in group A (Control). Laser irradiation parameters: wavelength-1064 nm, power density-0.75 W cm^{-2}, duration-6 min.

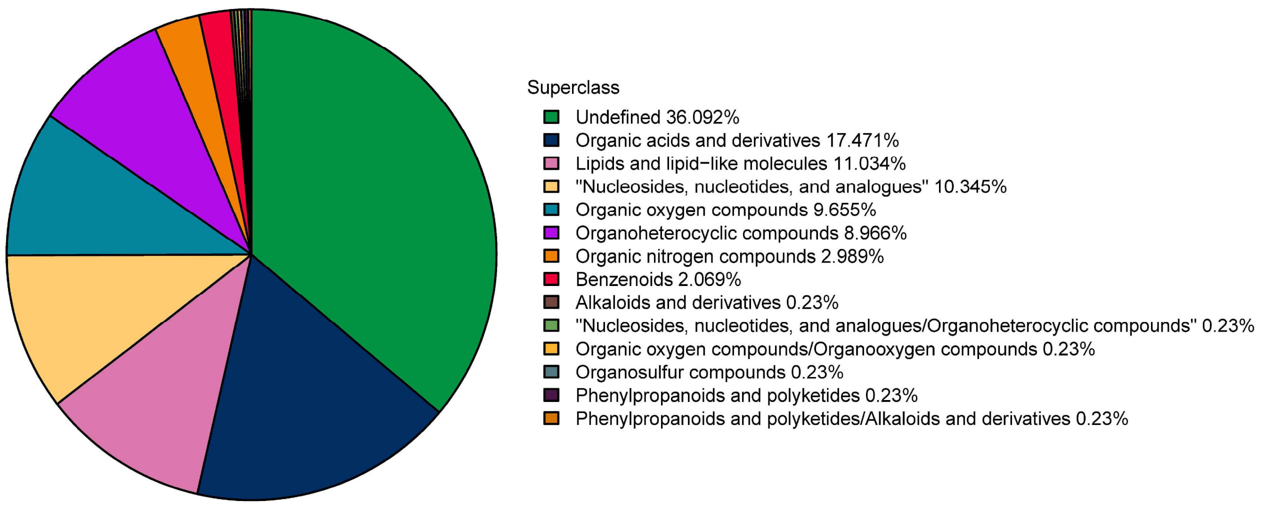

Figure S23. Quantitative proportions of identified metabolites in each chemical taxonomy. Note: The different color blocks represent different chemical classifications, and the percentage represents the proportion of those metabolites in the chemical classification attribution entry in all identified metabolites. Metabolites with no chemical classification are defined as undefined.

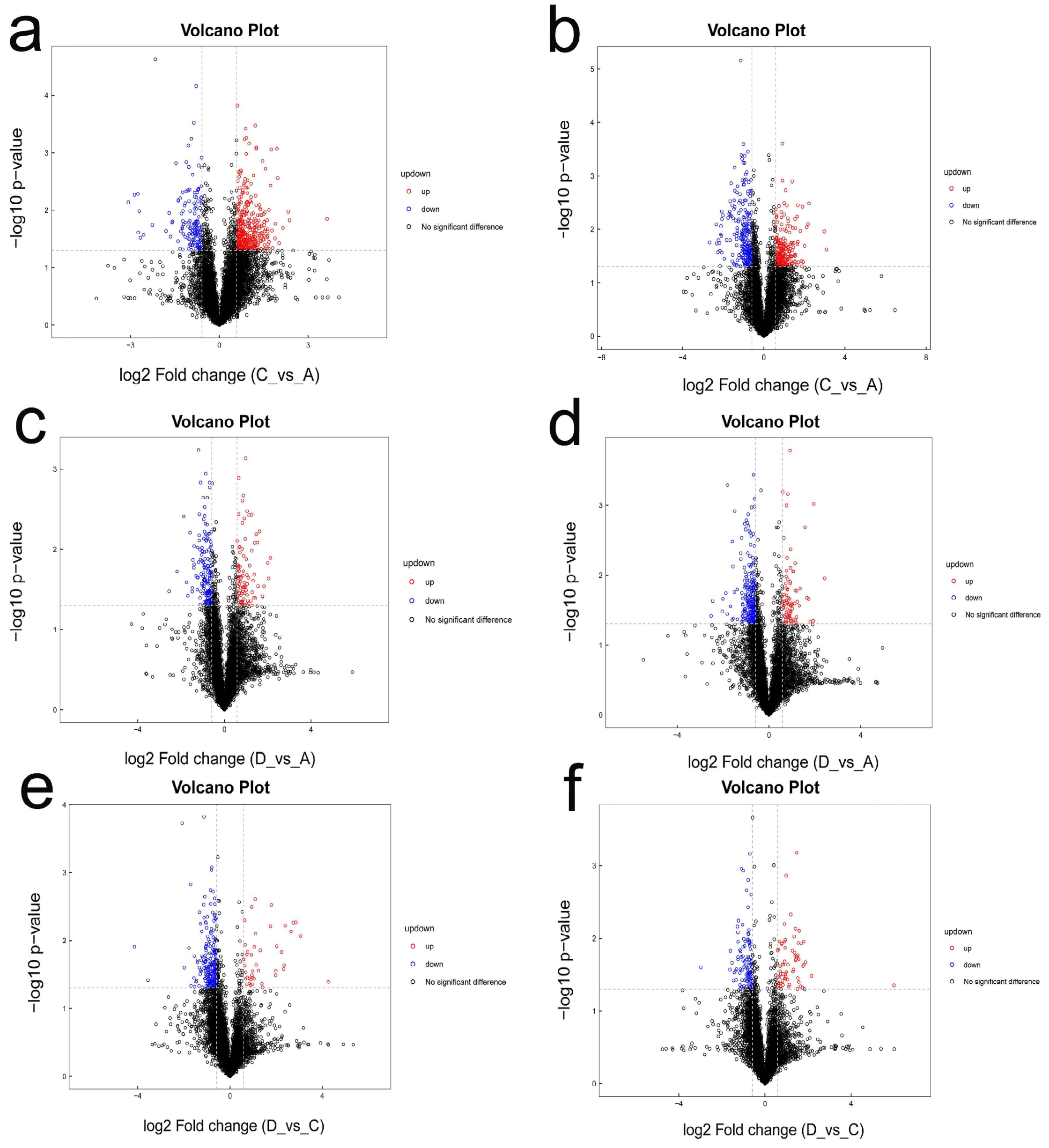

Figure S24. Volcano maps in POS (a,c,e) and NEG (b,d,f) ion modes with different comparisons between different two groups, *e.g.*, groups A and C (a,b), groups A and D (c,d) and groups C and D (e,f), where color difference correlates with the up- and down-regulation levels of metabolites. Based on univariate analysis, variance analysis of all metabolites (including unidentified metabolites) detected in positive and negative ion modes were performed. Differential metabolites with FC > 1.5 or FC < 0.67, P < 0.05, were visualized in their volcano plots, and the results are shown in Figure S16. Note: the horizontal axis of all graphs is log2 value of differential expression folds (Fold Change) and the vertical axis is log10 value of the significant P value. In those significantly-different metabolites: metabolites with FC > 1.5 and P < 0.05 are represented by red color; metabolites with FC < 0.67 and P < 0.05 are shown in blue; and non-significantly different metabolites are shown in black.

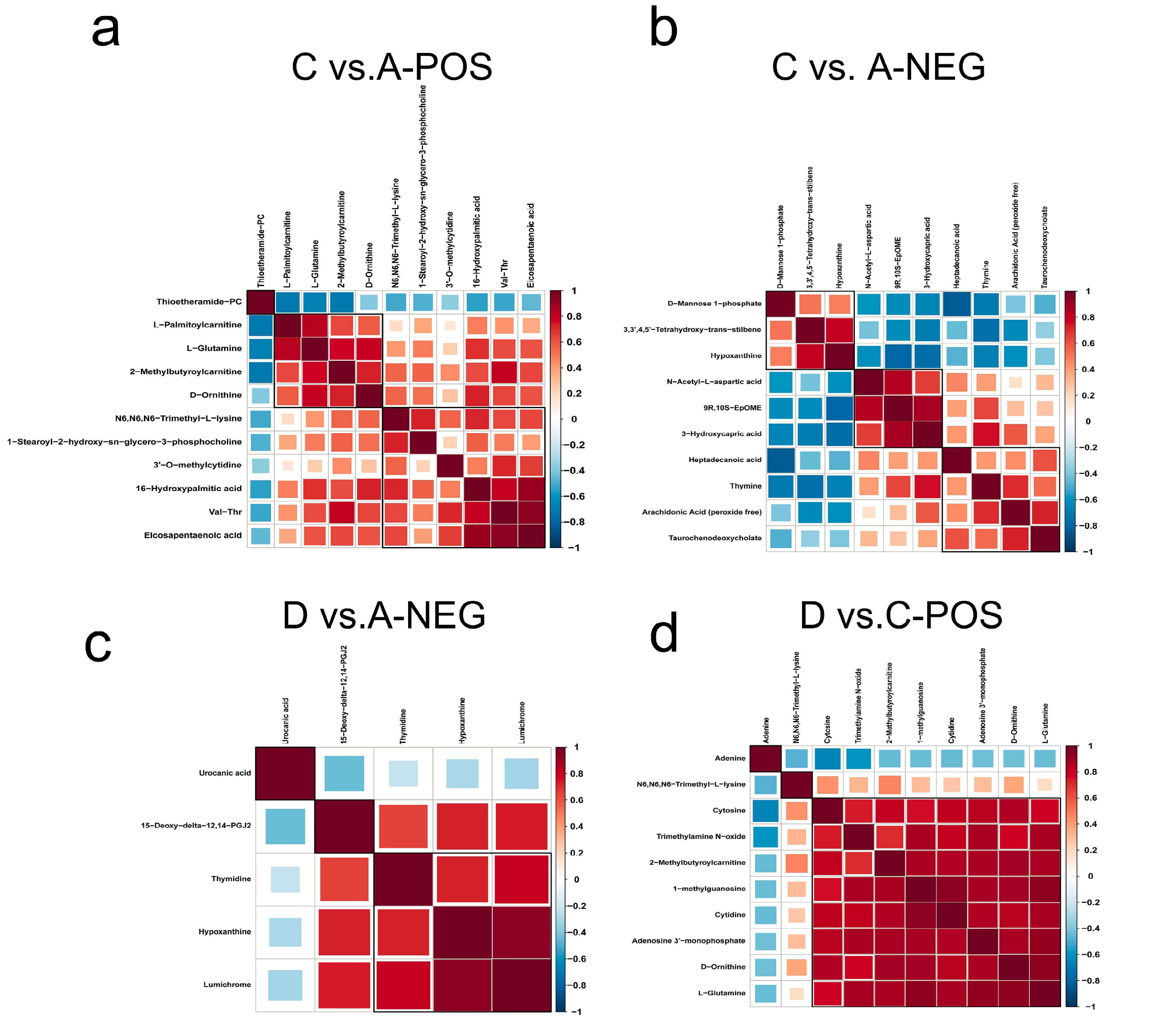

Figure S25 Heat maps of metabolite correlations in POS and/or NEG ion patterns for reflecting significant metabolite differences between different groups, *i.e.*, groups A and C (a,b), groups A and D (c) and groups C and D (d), where two comparisons (D vs. A-POS, and C vs. D-NEG) are missed due to the no significantly differential metabolites in their corresponding patterns according to the analysis (Figure 7). Note: red indicates positive correlation; blue indicates negative correlation; and white indicates non-significant correlation. The color shade is associated with the absolute size value of correlation coefficient, *i.e.*, the higher degree of positive or negative correlation corresponds to the darker color. The box size is related to the correlation significance, that is, the larger significance will result in the smaller p-value and the larger box. Notably, positive correlations indicated that the same metabolites originated from the same synthetic pathway, while negative correlations indicate that the same metabolites were obtained *via* catabolism of other metabolites, *i.e.*, synthetic transformation relationships.

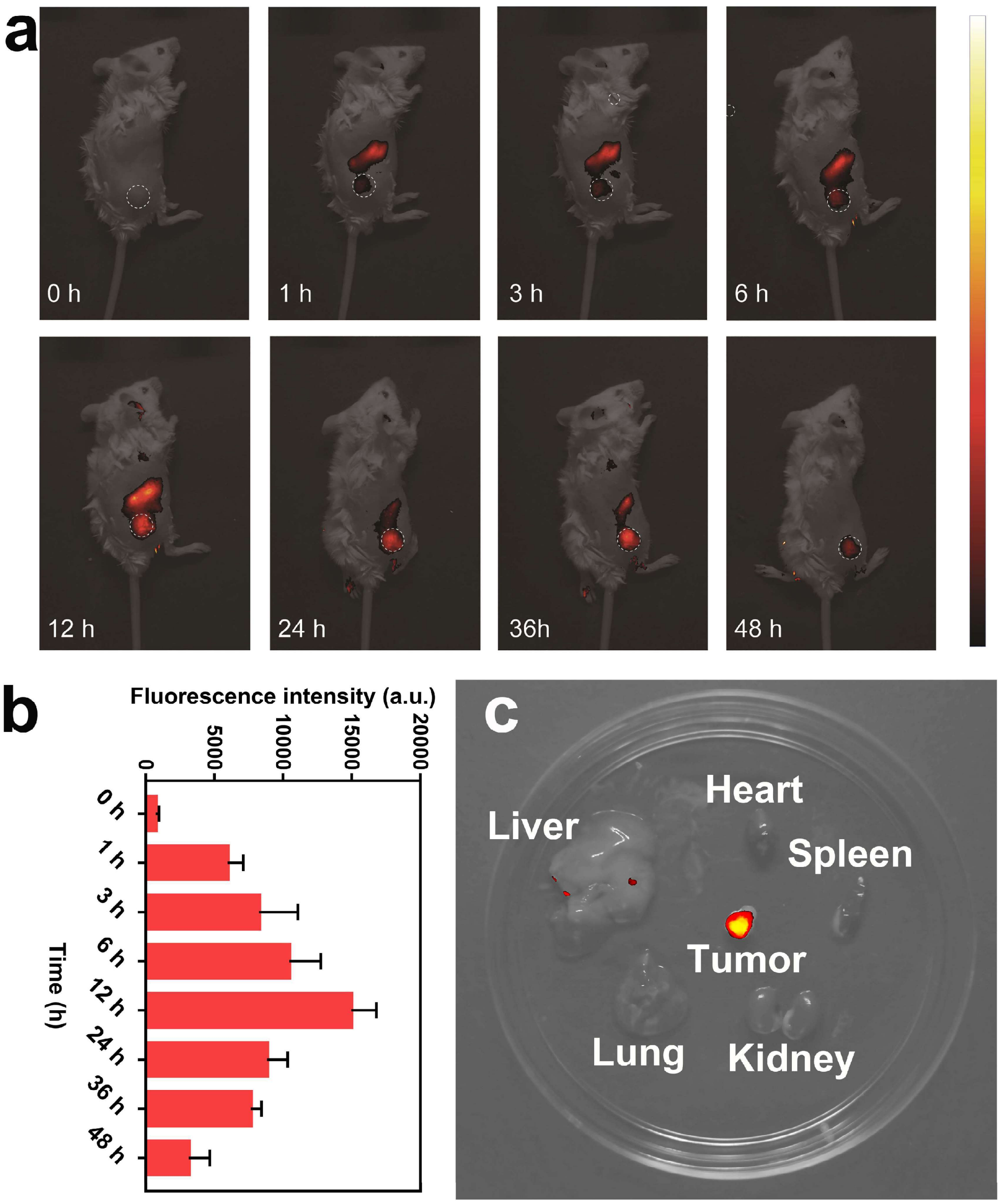

Figure S26. (a,b) Time-dependent *in vivo* fluorescence images (a) and corresponding fluorescence intensities (b) of CT26-bearing BALB/c mice that were intravenously injected with Nb_2C/Au/anti-TNFα-PVP at dosage of 4 mg/kg, wherein dotted circles indicate the tumor; (c) *Ex vivo* fluorescence images of normal organs and tumor harvested above mice. Data are expressed as mean ± SD (n=3).
